# A Theoretical Investigation on the Hydrogen Bond Based on the GLED Method of Bonding Analysis

**DOI:** 10.1002/jcc.70348

**Published:** 2026-03-09

**Authors:** Stefano Borocci, Felice Grandinetti, Nico Sanna, Costantino Zazza

**Affiliations:** ^1^ Dipartimento Per la Innovazione Nei Sistemi Biologici Agroalimentari e Forestali (DIBAF), Università Della Tuscia, L.go dell'Università Viterbo Italy; ^2^ Istituto Per i Sistemi Biologici del CNR Monterotondo RM Italy; ^3^ Istituto Per la Scienza e Tecnologia Dei Plasmi (ISTP) del CNR Bari Italy

**Keywords:** bonding analysis, electron energy density, GLED method, hydrogen bond

## Abstract

We propose here a description and classification of the hydrogen bond (HB) that is based on the Graphic representation of the Local electron Energy Density *H*(***r***) (GLED). A peculiar aspect of the GLED method, proposed by us in a recent study [*Journal of Chemical Physics* 163 (2025): 034107], is that the major character of the bond (covalent or noncovalent) can be inferred simply by the visual inspection of the plotted *H*(***r***), particularly the 3D *H*(***r***) = 0 isosurface. The analysis of the hydrogen‐bonded complexes unraveled, in particular, that their bonding character is strictly related to their dissociation energy (DE), so that the GLED assignment can be used to estimate the strength of the interaction. We also found that increasing values of DE mirror, in particular, an increased degree of covalency of the interaction. We could thus propose a classification of the HB that is based on the combined use of bonding character and stability. The HB was, in particular, assigned as weak (0.5–4.5 kcal mol^−1^), medium (3.5–5.5 kcal mol^−1^), and strong (4.5–15.0 kcal mol^−1^) for the neutral species, and medium (8.5–13.0 kcal mol^−1^), strong (15.0–32.0 kcal mol^−1^), and very strong (30.0–70.0 kcal mol^−1^) for the ionic ones, respectively. For systems stabilized by more than one HB, the method allows to eye‐catch in case different role of the various interactions.

## Graphical abstract

The 3D plot of the *H*(***r***) = 0 isosurface visually signals the noncovalent (nCov), weakly covalent (wCov), or polar covalent [Cov(pol)] character of neutral and ionic hydrogen‐bonded complexes. The increased degree of covalency mirrors an increased strength of the hydrogen bond (HB), which is classified based on the results of the bonding analysis.

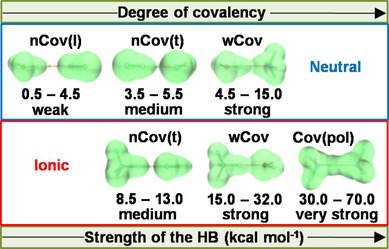

## 1 Introduction

The hydrogen bond (HB) is, arguably, the most extensively investigated intermolecular interaction, playing a major role in natural, biological, and synthetic processes [1, 2, 3, 4, 5, 6, 7, 8]. In its most classical formulation, also taken up in the IUPAC definition [9, 10], “the hydrogen bond is an attractive interaction between a hydrogen atom from a molecule or a molecular fragment X–H in which X is more electronegative than H, and an atom or a group of atoms in the same or a different molecule, in which there is evidence of bond formation”. In a hydrogen‐bonded complex X–H—Y–Z, X–H is the proton donor, and the proton acceptor Y may be an atom or an anion, or a fragment or a molecule Y–Z, where Y is bonded to Z. Over the years, as experimental and theoretical studies progressed, it emerged that the donors and the acceptors can be, actually, quite different in nature and type, so that the HB currently embraces a large variety of classical and nonclassical bonding motifs [11].

The HB is characterized in terms of different quantities, including geometric parameters [1, 5, 12, 13, 14, 15], spectroscopic absorptions in different spectral regions [9, 16, 17, 18, 19, 20, 21, 22, 23, 24, 25, 26, 27, 28, 29], and extent of proton transfer [30]. Particularly relevant is also the dissociation energy (DE), namely the energy demanded to separate the hydrogen‐bonded complex into its constituting fragments. For an assigned HB, the DE serves to rank the strength of the bond (e.g., weak, medium, or strong), and to infer suggestions about its nature. The nature and the DE of the HB are, indeed, intimately related, and the study of their relationships is a matter of great experimental and theoretical interest. While initially regarded as electrostatic in nature [31], the HB is, indeed, of complex character, having also contributions from dispersion, and from intra‐ and interfragments polarization, the latter eventually resulting in charge transfer and covalent contribution to the bond [9, 32]. This emerged, in particular, by the results of the theoretical calculations. The quantum theory of atoms in molecules (AIM) [33], the analysis of the electron localization function (ELF) [34, 35], of the reduced density gradient [36, 37], of the molecular electrostatic potential (MEP) [38, 39], and the natural bond orbital analysis [40] were already extensively employed to investigate the nature of the HB [41, 42, 43, 44, 45, 46, 47, 48, 49, 50, 51, 52, 53, 54, 55, 56, 57, 58, 59, 60]. Besides providing indications about the conceivable role of the various binding components, these studies unraveled quantitative correlations of predictive value between the DE and numerical indices such as the properties of the AIM bond critical point (BCP) [52], the core‐valence bifurcation index [61], and the ΔΔ*V*_*n*_ index defined by the MEP at nuclear positions [58]. Methods of energy decomposition analysis (EDA) [62, 63, 64, 65] are also quite informative about the nature of the HB [66, 67, 68]. They furnish, in fact, an explicit evaluation of the various binding components, making feasible their critical assay in complexes of different stability. Pursuing along this direction, Emamian, Lu, Kruse, and Emamian (ELKE) recently examined [69] 42 intermolecular hydrogen‐bonded dimers (henceforth denoted as the ELKE data set), judiciously chosen so to (i) include both neutral and ionic species, (ii) sample HBs involving various pairs of heteroatoms, and (iii) cover a wide range of stability of ca. 65 kcal mol^−1^. These systems were analyzed by symmetry‐adapted perturbation theory (SAPT) [65], whose results were compared with accurate DE values obtained at the coupled cluster level of theory including single and double excitations, and an estimated contribution of the triple, CCSD(T) [70]. For both the neutral and the ionic species, the magnitude of the DE resulted directly correlated with the dominating physical component(s), the correspondence being sufficiently strict to support a new classification of the HB. The neutral complexes were, thus, assigned as “very weak” or “weak‐to‐medium” HBs for DE lower than 2.5 kcal/mol or between 2.5 and 14.0 kcal/mol, respectively. The former are mainly dominated by dispersion together with electrostatics, the latter by electrostatics. The charged complexes were, instead, divided into “medium” or “strong” HBs for DE values in the range of 11.0–15.0 kcal/mol, or higher than 15.0 kcal/mol, respectively. The former are mainly dominated by electrostatics, and the latter by both electrostatics and induction. Based on the values of electron density *ρ*(***r***) at the BCP, *ρ*(BCP), and using linear interpolating equations strictly similar to those employed in previous studies [52], it was also possible to predict reliable values of the DE of both the neutral and the ionic interacting species. Overall, the ELKE study [69] shows that it is possible to accurately rank the strength of the HB based on the results of bonding analysis. This relationship between stability and bonding character should be valid also for systems larger than the model ones included in the ELKE data set. The costs of SAPT calculations are, however, rapidly increasing by increasing the molecular size, and it becomes, therefore, of interest to investigate whether accurate DE values of the HB could be anchored to bonding analyses performed by less expensive methods. A positive suggestion in this regard is obtained from the results of the present study, in which we examined the HB using our recently proposed method of bonding analysis [71]. It is based, essentially, on the Graphic representation of the Local electron Energy Density *H*(***r***) [72, 73, 74], and will be, therefore, from now referred to as the GLED method. Its major peculiarity is that the visual inspection of the plotted *H*(***r***) is in itself sufficient to describe the nature of the bond as covalent, noncovalent, or ionic. The assignment is then refined by calculating few numerical indexes obtained from the AIM analysis [33]. The computational costs are relatively low, and it is thus possible to examine systems of medium and large size. In particular, we studied here more than 80 hydrogen‐bonded systems, including the ELKE data set [69] and 40 species taken from the noncovalent interactions (NCI) Atlas data set [75, 76]. We could thus ascertain a strict correspondence between the assigned GLED nature of the various HBs and their strength, sufficient to rank and classify the bonds occurring in both neutral and ionic complexes. Equations are also proposed for more precise estimates of these values. We hope that our taken approach will actually find application in the study of different types of HB.

The paper is organized as follows. After providing essential computational details in Section 2, we recall in Section 3 the aspects of the GLED method that are most relevant to the present study, and perform the analysis of the species included in the ELKE data set [69]. We then discuss the GLED assignment of the additional species taken from the NCI Atlas data set [75, 76], and arrive to our proposed classification of the HB. We then examine further NCI Atlas complexes so to illustrate the conceivable use of our proposed method to analyze systems containing more than one HB. We finally report in Section 4 some concluding remarks, and considerations about the conceivable use of the GLED method to analyzed and classify other types of noncovalent interactions.

## 2 Computational Details

The calculations were performed at the density functional level of theory [77] using the B3LYP exchange‐correlation functional [78, 79] in conjunction with Grimme's D3(BJ) empirical dispersion correction [80]. The employed basis set was the ma‐TZVPP, which is the “minimally‐augmented” version of the def2‐TZVPP basis set [81, 82] for which *s* and *p* type diffuse basis functions are added to the nonhydrogen atoms. The B3LYP‐D3(BJ)/ma‐TZVPP wavefunctions of the investigated species were calculated with Gaussian 16 (Revision C.01) [83], using the B3LYP‐D3(BJ)/ma‐TZVPP or the B3LYP‐D3/def2‐QZVP optimized coordinates taken from the literature [69, 75, 76], and given in Data S1 and S2. The *ρ*(***r***) [33] and the *H*(***r***) [72, 73, 74] were analyzed using the Multiwfn program (version 3.8.dev) [84, 85]. Multiwfn [84, 85] was also employed to calculate the B3LYP‐D3(BJ)/ma‐TZVPP delocalization index *δ*(*X*,*Y*) in the AIM atomic space [86, 87, 88] of fragments *X* and *Y* forming complexes with covalent contribution, and to produce the three‐(3D) plots of the *H*(***r***) = 0 isosurfaces, henceforth denoted as *H*(0,ISO).

## 3 Results and Discussion

### 3.1 The GLED Method of Bonding Analysis

Within the GLED method [71], the bonding analysis of a molecular species is accomplished by examining the 2D and 3D plots of the *H*(***r***), and by computing a few numerical indices, namely the *ρ*(***r***) and the *H*(***r***) at the BCP located from the AIM analysis, and (for covalent species) the AIM bond delocalization index *δ*. The full details are given in reference [71], and in other strictly related studies [89, 90, 91, 92, 93, 94]. We briefly recall here the aspects that are most relevant to the present investigation.

**FIGURE 1:**
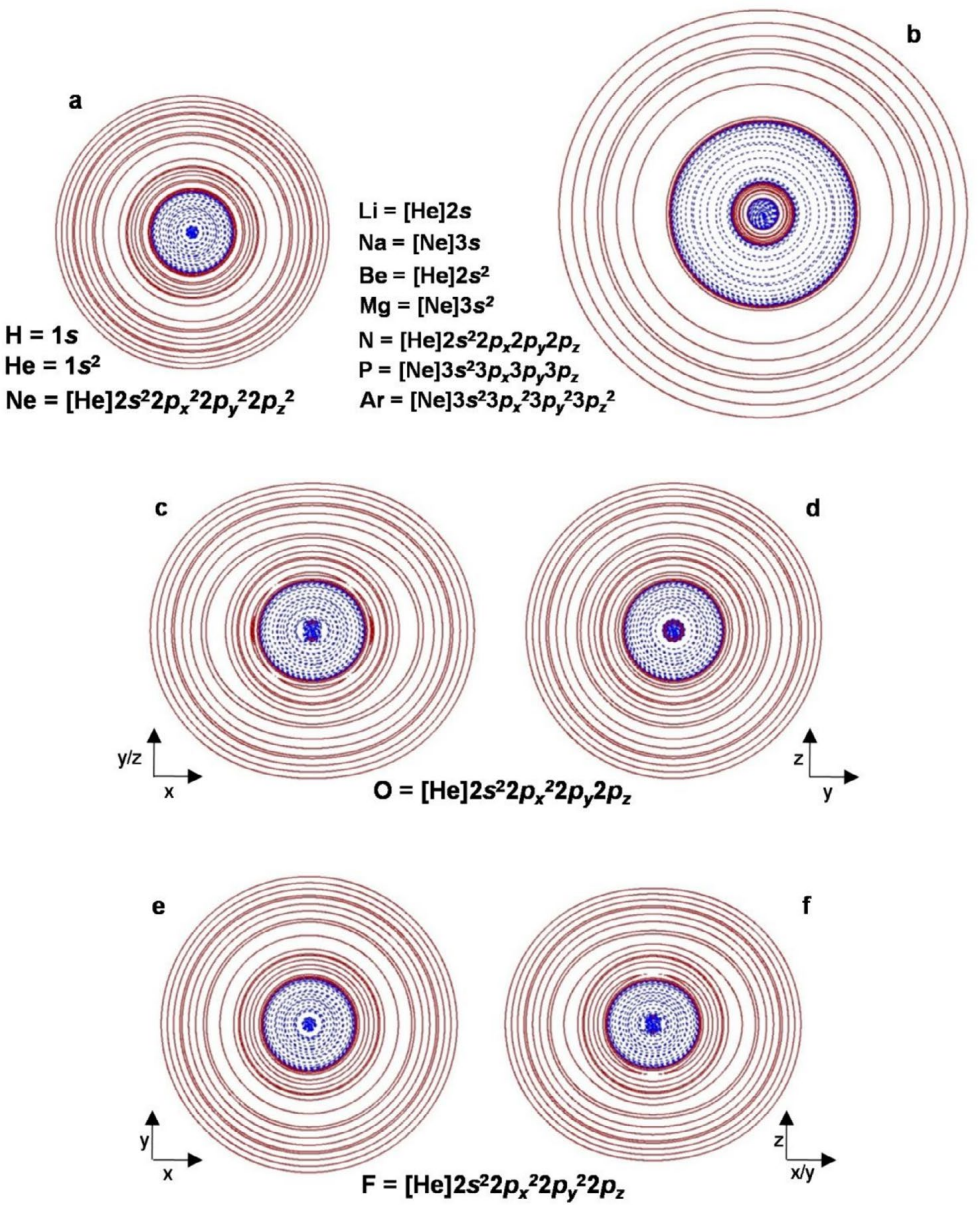
2D‐plots of the *H*(***r***) of (a) H, He, Ne, (b) Li, Na, Be, Mg, N, P, Ar, (c, d) O, and (e, f) F atoms showing the partition of the space into *H* ^−^(***r***) (dashed/blue lines) and *H* ^+^(***r***) regions (solid/brown lines). For atoms featuring four regions in the sequence *H* ^−^(***r***)/*H* ^+^(***r***)/*H* ^−^(***r***)/*H* ^+^(***r***), the populations of the inner and outer *H* ^−^(***r***)/*H* ^+^(***r***) pairs account, respectively, for the core (*ca*. 2*e* for the first‐row and *ca*. 10*e* for second‐row elements, respectively) and the valence electrons.

**FIGURE 2:**
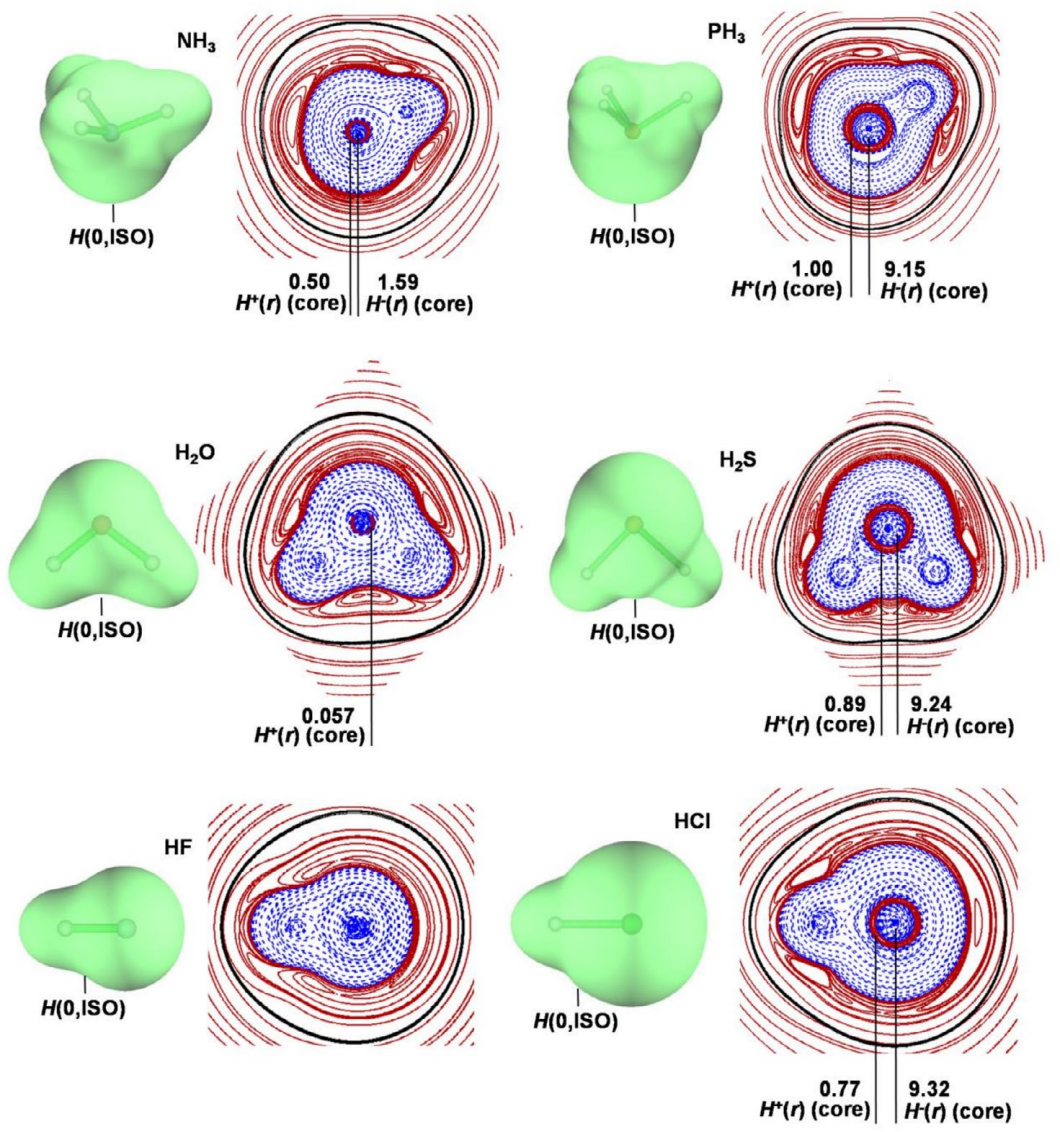
3D‐plots of the *H*(0,ISO) and 2D‐plots of the *H*(***r***) in the main plane of the 10‐electrons covalent hydrides, NH_3_, PH_3_, H_2_O, H_2_S, HF, and HCl (the solid/brown and dashed/blue lines correspond, respectively, to positive and negative values). The quoted numbers are the population (*e*) of the atomic core domains (MP2/aug‐cc‐pVTZ values taken from the reference [71]), and the black line corresponds to the van der Waals surface (*ρ* = 0.0010*e a*_0_^−3^).

The GLED method takes advantage of a major property of the *H*(***r***), namely its partitioning of the atomic space into regions of negative values, indicated here as *H* ^−^(***r***), which alternate with regions of positive values, indicated here as *H* ^+^(***r***). By integrating the *ρ*(***r***) over these regions, it becomes possible to evaluate how many electrons in an atom (formally) possess negative or positive energies. The 2D plots of the *H*(***r***) of the first 18 elements H–Ar are shown in Figures 1S and 2S, and some exemplary situations are also given in Figure 1. The relevant quantities are reported in Table 1. H, He, and Ne feature just two regions (Figure 1a), an inner *H* ^−^(***r***) hosting the majority of the electrons (0.79*e*, 1.40*e*, and 8.96*e*, respectively), and an outer *H* ^+^(***r***). Their spherical shape mirrors the symmetric electronic configuration of the atoms. A wide *H* ^−^(***r***) region hosting both core and valence electrons, with populations of 7.05*e* and 8.07*e*, respectively, is also occurring in the atomic space of O (Figure 1c,d) and F (Figure 1e,f), which as well includes a valence *H* ^+^(***r***) with a population of 0.79*e* and 0.905*e*, respectively, and a quite small core *H* ^+^(***r***) with a population of 0.16*e* and 0.025*e*, respectively. The atomic space of all the other elements is, instead, partitioned into four regions of spherical (Li, Na, Be, Mg, N, P, and Ar) (Figure 1b) or nonspherical shape (B, Al, C, Si, S, and Cl) (Figures 1Sc–f and 2Sc,d,g,h), two *H* ^−^(***r***), and two *H* ^+^(***r***), which alternate from an innermost *H* ^−^(***r***) to an outermost *H* ^+^(***r***) in the sequence *H* ^−^(***r***)/*H* ^+^(***r***)/*H* ^−^(***r***)/*H* ^+^(***r***). Interestingly, the inner and outer *H* ^−^(***r***)/*H* ^+^(***r***) pairs are clearly referable, respectively, to the core and valence electrons of the various atoms. The populations of the former are, in fact, invariably predicted (see Table 1) as *ca*. 2*e* for the first‐row and *ca*. 10*e* for second‐row elements, and the populations of the latter overall account for the valence electrons of the various atoms.

**TABLE 1:** Occupations (*e*) of the *H* ^−^(***r***) and *H* ^+^(***r***) regions of H–Ar (see Figures 1, S1, and S2) (MP2/aug‐cc‐pVTZ values taken from the reference [71]).

Atom	*H* ^−^(*r*)	*H* ^+^(*r*)
H	0.79	0.21
He	1.40	0.60
Ne	8.96	1.04

Atom	*H* ^−^(*r*)	*H* ^+^(*r*) (core)	*H* ^+^(*r*) (valence)
O	7.05	0.16	0.79
F	8.07	0.025	0.905

Atom	*H* ^−^(*r*) (core)	*H* ^+^(*r*) (core)^a^	*H* ^−^(*r*) (valence)	*H* ^+^(*r*) (valence)
Li	1.49	0.58 (2.07)	0.72	0.21
Be	1.54	0.56 (2.10)	1.48	0.42
B	1.54	0.56 (2.10)	2.39	0.51
C	1.58	0.51 (2.09)	3.32	0.59
N	1.64	0.39 (2.03)	4.28	0.69
Na	8.86	1.30 (10.16)	0.65	0.19
Mg	8.93	1.31 (10.24)	1.35	0.41
Al	8.98	1.25 (10.23)	2.19	0.58
Si	9.04	1.13 (10.17)	3.11	0.72
P	9.15	0.99 (10.14)	4.10	0.76
S	9.23	0.89 (10.12)	5.02	0.86
Cl	9.32	0.77 (10.09)	5.94	0.97
Ar	9.42	0.61 (10.03)	6.86	1.11

^a^ The value in parentheses is *H* ^−^(***r***) (core) + *H* ^+^(***r***) (core).

When the atoms form chemical bonds, their *H* ^−^(***r***)/*H* ^+^(***r***) core regions remain, essentially, unperturbed, but their *H* ^−^(***r***)/*H* ^+^(***r***) valence regions, and their hosted electrons, combine in different modes; any formed bond is assigned as covalent, noncovalent, or ionic by visualizing, in particular, the modes of combination of the *H* ^−^(***r***) regions, and by comparing the electronic populations of the combined regions with those of the precursor atoms. Taking into account the matter discussed in this study (*vide infra*), we illustrate here, in particular, the features signing the formation of covalent bonds of different character, and of noncovalent bonds. A covalent bond is visually caught as the *overlapping of the valence H* ^*−*^ *(**r**) domains of the involved atoms, so to form a Valence H* ^*−*^ *(**r**) Binding Domain (henceforth denoted as VBD) that encloses the valence electrons with negative energies*. In this process, the number of electrons having negative energies remains, essentially, unchanged. The VBD is plunged into an outer *H* ^+^(***r***) region, and these two regions are delimited by a *H*(0,ISO) enclosing the nuclei of the atoms forming the bond. *The occurrence of such H(0,ISO) is, indeed, the GLED signature of the covalent bond*. Typical illustrative examples are the 10‐ and 18‐electrons hydrides NH_3_, PH_3_, H_2_O, H_2_S, HF, and HCl, typically also involved in HB. Their *H*(***r***) plots are shown in Figure 2. One first notes that in all these molecules the core electrons remain, essentially, unperturbed. This is in line with studies based, for example, on the ELF approach [34, 35, 95], showing that core electrons in various reactions and processes practically do not change. As for the valence electrons, they actually produce the fingerprint of covalency, namely a *H*(0,ISO) encompassing the nuclei of the bound atoms (visible in the 3D plots), and enclosing the VBD formed by the overlapped electrons with negative energies (best viewed in the 2D plots). As shown in Table 2, the populations of these regions are, essentially, unaffected with respect to the total number of valence electrons of the separated atoms having negative energy. Once generally assigned as covalent, the detailed nature of the bond may range from homopolar or nearly homopolar to strongly heteropolar. We thus classified the covalent bonds based on the combined use of the *ρ*(BCP) and the AIM delocalization index *δ*(*X*,*Y*) [86, 87, 88]. The latter measures, in particular, the number of electrons that are exchanged between *X* and *Y* [86], and gives, in essence, the fractional number of electron pairs shared by the two atoms [87, 88]. As discussed previously [86, 87], covalent bonds with values of *δ*(*X*,*Y*) significantly lower than 1 mirror appreciably different atomic charges, and the polar nature of the *X*–*Y* interaction.

**TABLE 2:** Occupations of the valence *H* ^−^(***r***) binding domains n(VBD) (*e*) of the covalent hydrides of the first‐ and second‐row nonmetal elements (MP2/aug‐cc‐pVTZ values taken from reference [71]).

	*n*(VBD)^a^	*ρ*(BCP)^b^	*δ*(*X*,*Y*)^c^	Assignment
NH_3_	6.78 (6.65)	0.3420	0.862	Cov
PH_3_	6.51 (6.47)	0.1632	0.805	Cov
H_2_O	8.80 (8.63)	0.3639	0.593	Cov(pol)
H_2_S	6.65 (6.60)	0.2207	1.150	Cov
HF	8.91 (8.86)	0.3660	0.373	Cov(pol)
HCl	6.76 (6.73)	0.2536	0.976	Cov

^a^ The value in parenthesis is the total number of valence electrons of the separated atoms having negative energy.

^b^ Electron density (*e a*_0_^−3^) at the BCP.

^c^ AIM delocalization index (*e*) (HF/aug‐cc‐pVTZ values taken from the reference [71]).

Based, in particular, on the *ρ*(BCP) and *δ*(*X*,*Y*) predicted for a large ensemble of representative species [71], we proposed the following classification: (a) a bond is assigned as covalent (Cov) if *δ*(*X*,*Y*) ≥ 0.8*e*, irrespective of *ρ*(BCP); (b) a bond is assigned as polar covalent [Cov(pol)] or partially covalent (pCov) if 0.2*e* ≤ *δ*(*X*,*Y*) < 0.8*e*, and if *ρ*(BCP) ≥ 0.05*e a*_0_^−3^, or *ρ*(BCP) < 0.05*e a*_0_^−3^, respectively; (c) a bond is assigned as weakly covalent (wCov) if *δ*(*X*,*Y*) < 0.2*e*, irrespective of *ρ*(BCP). The bonds occurring in the hydrides shown in Figure 2 are, in particular, assigned as shown in Table 2. We note in passing that, while the delocalization index is sensitive to binding changes (cov or cov(pol)), the electron density at BCP is less affected. This further supports the combined use of these two indices to assign the bonding character.

**FIGURE 3:**
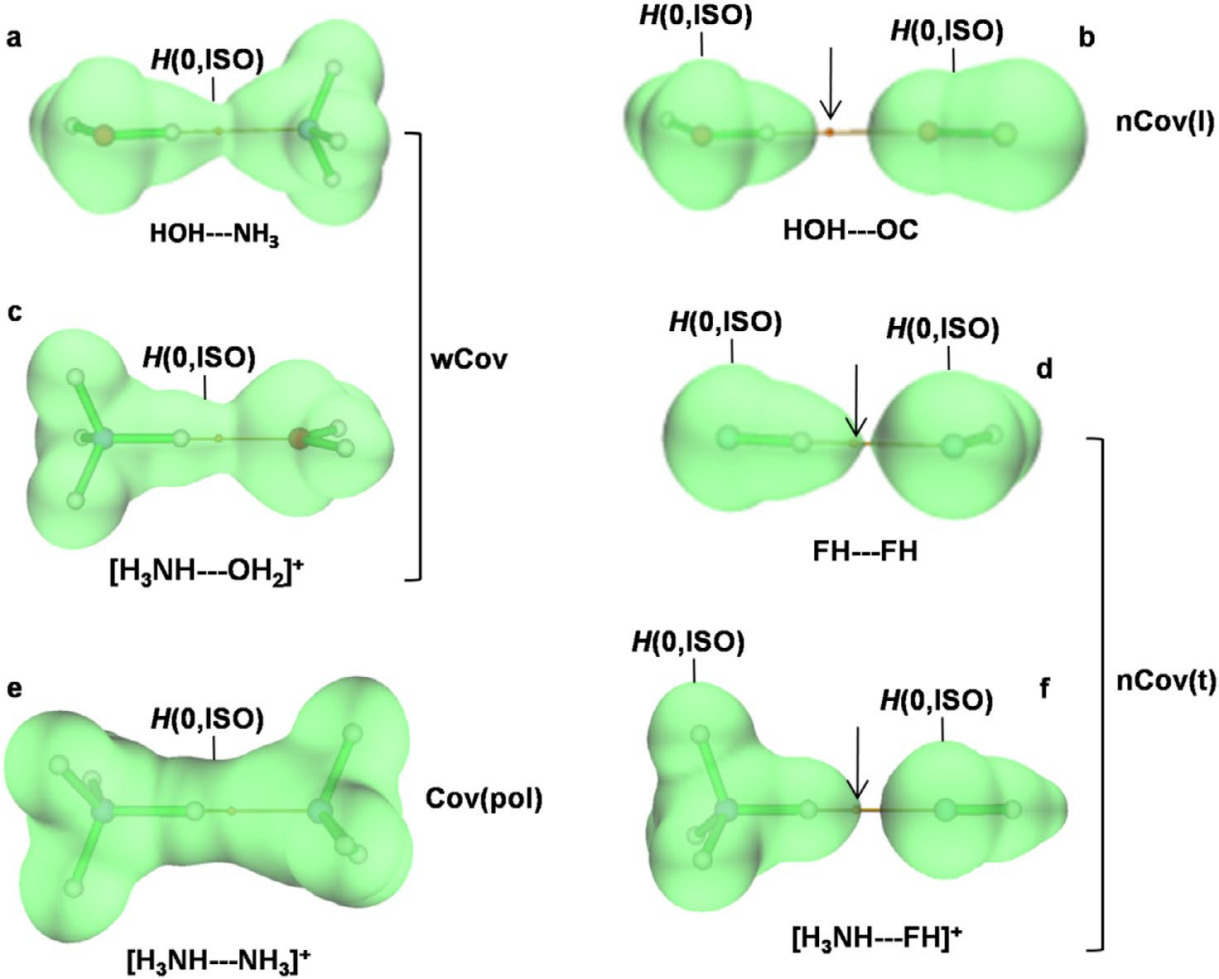
3D‐plots of the *H*(0,ISO) of the (a) HOH—NH_3_, (b) HOH—OC, (c) [H_3_NH—OH_2_]^+^, (d) [FH—FH], (e) [H_3_NH—NH_3_]^+^, and (f) [H_3_NH—FH]^+^ hydrogen‐bonded complexes taken from the reference [69] that are exemplary of the GLED assignment of different types of intermolecular interactions.

The contacts between molecules stabilized by covalent bonds of different nature generally produce intermolecular complexes of different bonding character. The GLED method furnishes an effective description of the various situations, illustrated here by discussing some exemplary cases of HB taken from the ELKE data set [69] and reported in Figure 3.

For systems such as HOH—NH_3_ (Figure 3a), [H_3_NH—OH_2_]^+^ (Figure 3c), and [H_3_NH—NH_3_]^+^ (Figure 3e), the bonding mechanism is qualitatively similar to that occurring in the formation of covalent molecules from interacting atoms. In each of these complexes, in fact, the two fragments overlap so to produce an outer *H*(0,ISO) encompassing the nuclei of both fragments, and enclosing the electrons with negative energies which populate the VBD of their precursor moieties. The *ρ*(BCP)/*δ*(X,Y) of HOH—NH_3_, [H_3_NH—OH_2_]^+^, and [H_3_NH—NH_3_]^+^ are, in particular, 0.0305*e a*_0_^−3^/0.098*e*, 0.0545*e a*_0_^−3^/0.139*e*, and 0.0863*e a*_0_^−3^/0.241*e*, respectively, and the corresponding OH—N, NH—O, and NH—N bonds are, therefore, assigned as wCov, wCov, and Cov(pol), respectively.

As shown in Figure 3b,d,f, for systems such as HOH—OC, F–H—FH, and [H_3_NH–FH]^+^, the composing fragments overlap their outermost *H* ^+^(***r***) regions, but *their valence H(0,ISO) do not touch each other, and their enclosed valence H* ^*−*^ *(***r***) regions remain separate*. This separation of the *H*(0,ISO) is, indeed, the GLED visual signature of complexes of noncovalent (nCov) character. In these systems, the interacting fragments do not share the electrons with negative energies, and the *n*(VBD) of the composing moieties remain, essentially, unchanged when they form the complex. The effective use of the *H*(0,ISO) in the context of the study of NCIs, not only HBs, was already highlighted in previous studies. For example, the plotted *H*(***r***) = 0 “reactive surfaces” were effectively employed to analyze and distinguish systems stabilized by strong and weak triel bonds [96]. One also notes from Figure 3b,d,f that, along the bond path (BP) connecting the interacting atoms, the *H*(***r***) is null at the two *H*(0,ISO) of the interacting fragments, is negative inside their VBD, and is positive in their separating *H* ^+^(***r***) region. Thus, depending on the position of the BCP along the BP, the *H*(BCP) may be positive, negative or null. We thus distinguished the nCov interactions into *loose*, nCov(l), or *tight*, nCov(t), if *H*(BCP) ≥ 0 or *H*(BCP) < 0, respectively. The *H*(BCP) of HOH—OC is 0.0020 *hartree a*_0_^−3^, and the OH—O contact is, therefore, assigned as nCov(l). The *H*(BCP) of both F–H—FH and [H_3_NH–FH]^+^ is negative (−0.0023 and −0.0014 *hartree a*_0_^−3^, respectively), and these contacts are, instead, assigned as nCov(t).

In summary, the GLED method allows a graphical study of the nature of the chemical bond, based on the 2D and 3D representation of the *H*(***r***). Particularly, important is the 3D visualization of the *H*(0,ISO), which in itself is sufficient to indicate the covalent or noncovalent nature of the interaction. The bond is then further characterized in terms of numerical indices typical of the AIM analysis [33], such as the *ρ*(BCP), the *H*(BCP), and the delocalization index *δ*(*X*,*Y*). In the forthcoming paragraphs, we discuss the application of the method to the study of the nature and the strength of the HB.

### 3.2 The GLED Analysis of the ELKE Data Set

To explore the description of the HB furnished by the GLED method, and to eventually arrive at a classification of the HB (*vide infra*), we first analyzed the 42 species (28 neutral and 14 cationic) included in the ELKE data set [69]. They are listed in the second column of Table 3. Using the B3LYP‐D3(BJ)/ma‐TZVPP geometries reported in the ELKE study [69], we performed the GLED analysis at the same computational level. The obtained results are listed in the third column of Table 3. Eighteen neutral complexes were assigned as nCov(l), three were assigned as nCov(t), and seven were assigned as wCov. As for the ionic complexes, two were assigned as nCov(t), six were assigned as wCov, and six were assigned as Cov(pol). Interestingly, as shown in the fourth column of Table 3, when these different bonding characters are grouped in the order nCov(l)/nCov(t)/wCov, and nCov(t)/wCov/Cov(pol) for the neutral and the ionic complexes, respectively, this produces a nearly perfect rank of the species by increasing values of their CCSD(T)/jul‐cc‐pVTZ DEs [69].

**TABLE 3:** GLED assignment, dissociation energy DE (kcal mol^−1^), electron density *ρ* (*e a*_0_^−3^), and energy density *H* (*hartree a*_0_^−3^) of the hydrogen‐bonded complexes included in the ELKE data set [69].

Type of complex	Structure	GLED assignment^a^	DE^b^	*ρ*(BCP)^c^	*H*(BCP)^c^
Neutral	H_3_CH—NCH	nCov(l)	0.60	0.0047	0.0010
	H_3_CH—OH_2_	nCov(l)	0.62	0.0064	0.0012
	MeCCH—OC	nCov(l)	0.68	0.0055	0.0015
	H_3_CH—NH_3_	nCov(l)	0.71	0.0069	0.0010
	HCCH—OC	nCov(l)	0.73	0.0059	0.0016
	FCCH—OC	nCov(l)	0.78	0.0059	0.0016
	HOH—OC	nCov(l)	0.97	0.0088	0.0020
	HSH—SH_2_	nCov(l)	1.51	0.0113	0.0008
	HCCH—SH_2_	nCov(l)	1.54	0.0087	0.0009
	HSH—N_3_H	nCov(l)	2.10	0.0146	0.0012
	HSH—OH_2_	nCov(l)	2.58	0.0161	0.0016
	HOH—SH_2_	nCov(l)	2.78	0.0158	0.0001
	HCCH—OH_2_	nCov(l)	2.83	0.0145	0.0020
	N_3_H—FH	nCov(l)	2.94	0.0157	0.0025
	H_2_NH—NH_3_	nCov(l)	3.03	0.0160	0.0011
	N_3_H—SH_2_	nCov(l)	3.16	0.0167	0.0002
	HSH—NH_3_	nCov(l)	3.34	0.0213	0.0001
	HCCH—NH_3_	nCov(l)	3.56	0.0165	0.0010
	HOH—N_3_H	nCov(t)	3.73	0.0221	−0.0006
	FH—FH	nCov(t)	4.52	0.0281	−0.0023
	N_3_H—OH_2_	nCov(t)	5.43	0.0258	−0.0005
	HOH—OH_2_	wCov (0.075)	4.93	0.0259	−0.0014
	FH—SH_2_	wCov (0.101)	5.06	0.0274	−0.0041
	HOH—NH_3_	wCov (0.098)	6.41	0.0305	−0.0035
	FH—N_3_H	wCov (0.087)	7.09	0.0399	−0.0087
	N_3_H—NH_3_	wCov (0.120)	7.54	0.0337	−0.0039
	FH—OH_2_	wCov (0.094)	8.89	0.0450	−0.0112
	FH—NH_3_	wCov (0.136)	13.36	0.0585	−0.0201
Ionic	[H_2_SH—FH]^+^	nCov(t)	11.27	0.0351	−0.0032
	[H_3_NH—FH]^+^	nCov(t)	12.24	0.0320	−0.0014
	[HOH—SH]^−^	wCov (0.146)	15.14	0.0297	−0.0041
	[H_2_NH—OH]^−^	wCov (0.180)	16.94	0.0539	−0.0142
	[H_2_NH—F]^−^	wCov (0.164)	17.34	0.0562	−0.0148
	[H_2_OH—FH]^+^	wCov (0.117)	19.29	0.0662	−0.0224
	[H_3_NH—OH_2_]^+^	wCov (0.139)	20.91	0.0545	−0.0138
	[HOH—F]^−^	wCov (0.188)	31.67	0.0930	−0.0458
	[H_3_NH—NH_3_]^+^	Cov(pol) (0.241)	31.46	0.0863	−0.0398
	[HOH—OH]^−^	Cov(pol) (0.254)	37.17	0.1164	−0.0728
	[H_2_OH—SH_2_]^+^	Cov(pol) (0.377)	37.73	0.0988	−0.0579
	[Cl—H—Cl]^−^	Cov(pol) (0.451)	42.70	0.1172	−0.0741
	[H_2_O—H—OH_2_]^+^	Cov(pol) (0.269)	52.74	0.1703	−0.1934
	[F—H—F]^−^	Cov(pol) (0.254)	65.47	0.1783	−0.2314

^a^ B3LYP‐D3(BJ)/ma‐TZVPP calculations at the B3LYP‐D3(BJ)/ma‐TZVPP optimized geometries taken from the reference [69]. For the HBs of covalent character, the value in parentheses is the B3LYP‐D3(BJ)/ma‐TZVPP AIM delocalization index.

^b^ CCSD(T)/jul‐cc‐pVTZ//B3LYP‐D3(BJ)/ma‐TZVPP values with half counterpoise correction taken from the reference [69].

^c^ Calculated at the B3LYP‐D3(BJ)/ma‐TZVPP AIM BCP.

**FIGURE 4:**
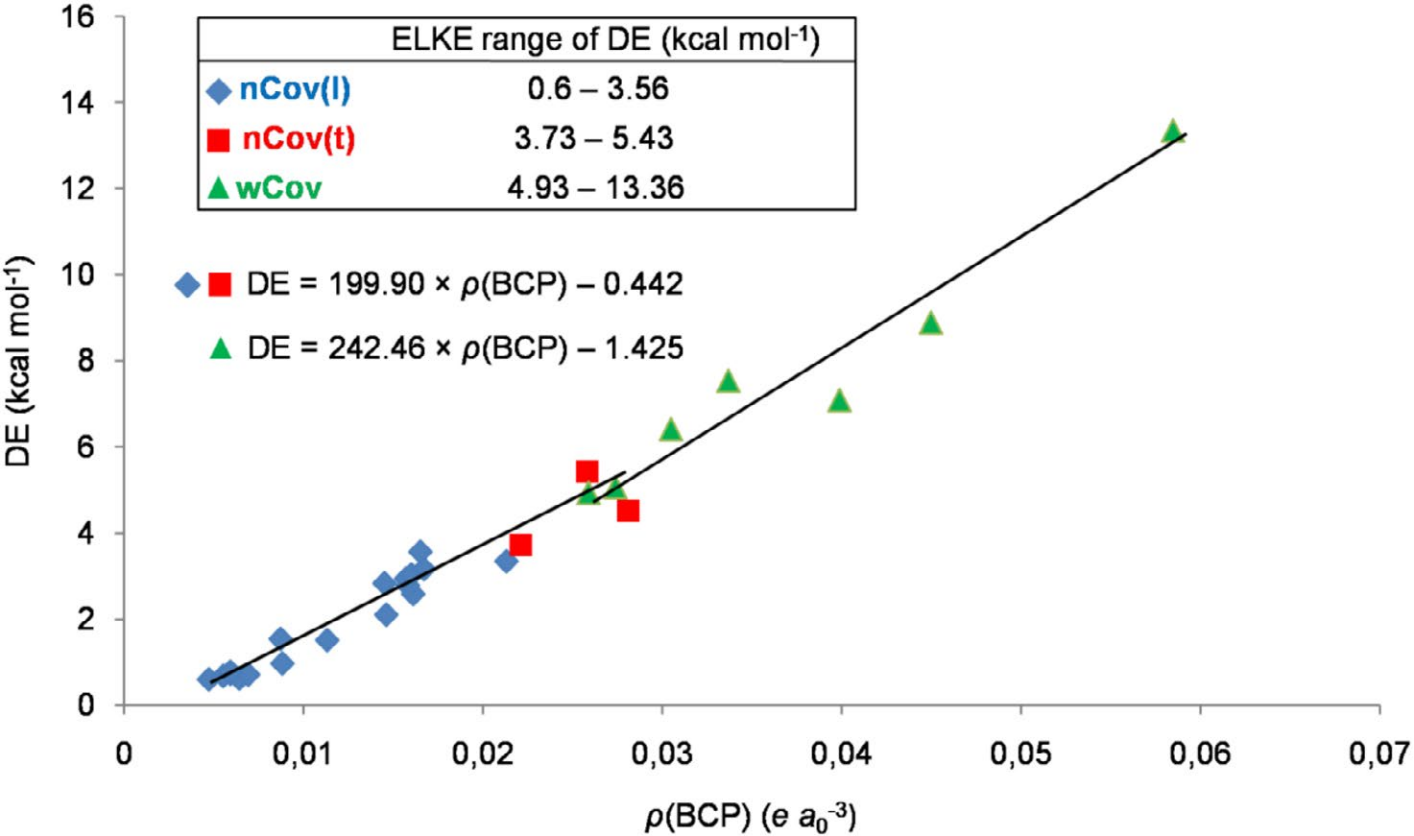
Regression plot of DE versus *ρ*(BCP) of the neutral complexes included in the ELKE data set [69].

**FIGURE 5:**
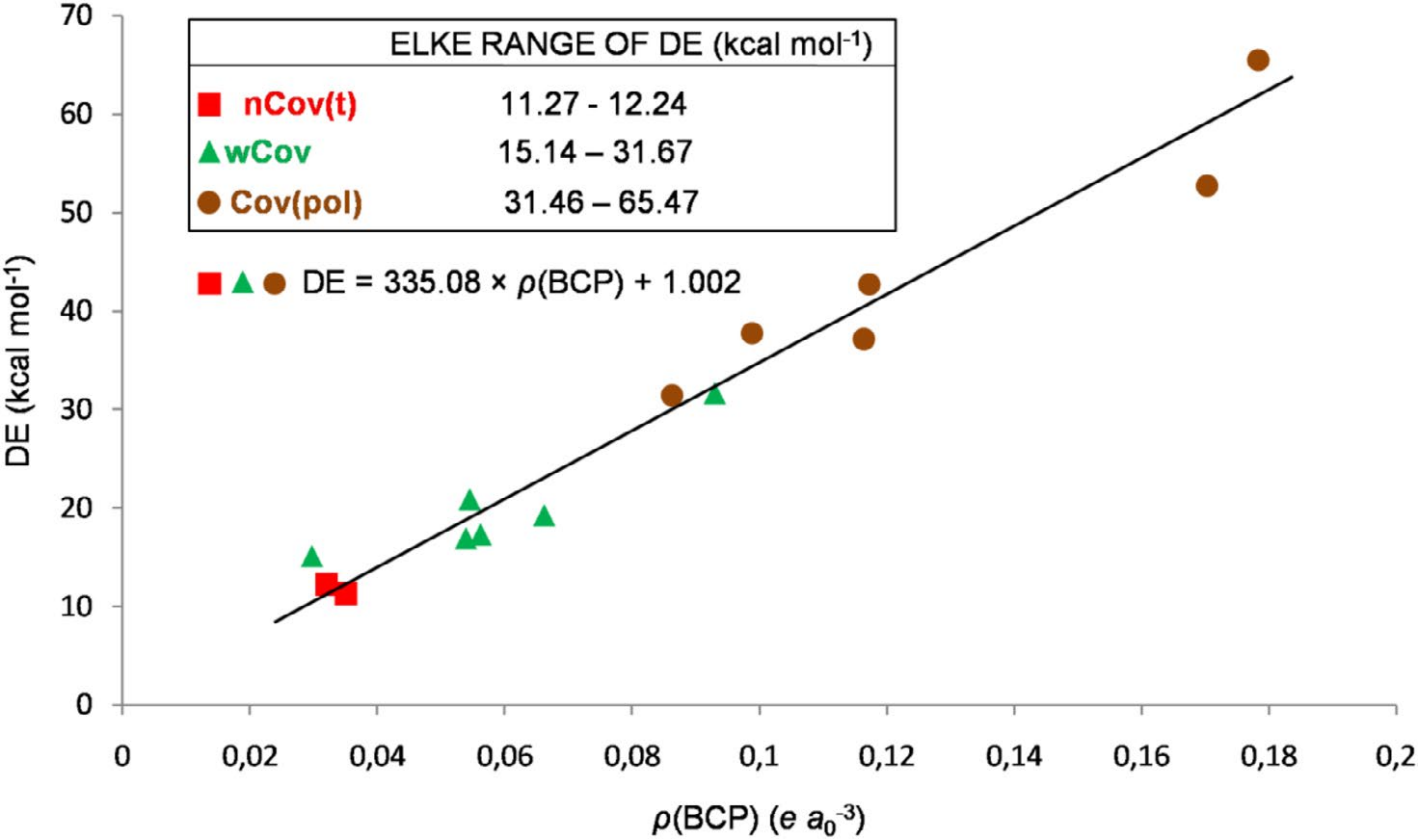
Regression plot of DE versus *ρ*(BCP) of the ionic complexes included in the ELKE data set [69].

Thus, the GLED method effectively catches the progressively variable nature of the HB that accompanies the increase in the extent of the interaction, in particular, the tendency for the inductive component (intrafragment polarization and/or electron sharing) to increase its contribution when the stability of the complex tends to increase [69]. This gradually varying nature of the HB is also visually caught when looking at the graphs reported in Figures 4 and 5, showing, respectively, how the neutral and the ionic ELKE complexes populate the *ρ*(BCP)/DE plane as a function of their bonding character. The values of the DE and the *ρ*(BCP) of the ELKE complexes are, indeed, strictly correlated [69]. Using two distinct linear equations for the neutral and the ionic species, it was possible [69] to use the *ρ*(BCP) so to predict the DE with a mean absolute percentage error (MAPE) of 14.69% and 10.03%, respectively. In the present study, we preferred to interpolate the data of the neutral complexes using two distinct equations for the nCov(l)/nCov(t) and the wCov ones, which predict the DE values with a MAPE of 14.32% (*R* ^2^ = 0.934) and 7.09% (*R* ^2^ = 0.941), respectively:

(1) $$\mathrm{DE}\left(n/\text{nCov}\right)=199.90\times \rho \left(\mathrm{BCP}\right)-0.442$$

(2) $$\mathrm{DE}\left(n/\text{wCov}\right)=242.46\times \rho \left(\mathrm{BCP}\right)-1.425$$

The single equation used to interpolate the data of the ionic species predicts the DE values with a MAPE of 10.77% (*R* ^2^ = 0.962) and is, essentially, coincident with that employed in the ELKE study [69]:

(3) $$\mathrm{DE}\left(i\right)=335.08\times \rho \left(\mathrm{BCP}\right)+1.002$$

Overall, the present analysis unraveled a relationship between the GLED bonding character of the ELKE species and their stability. In particular, based on the data quoted in Table 3, neutral complexes assigned as nCov(l), nCov(t), and wCov are expected to feature DE values in the range 0.60–3.56, 3.73–5.43, and 4.93–13.36 kcal mol^−1^, respectively, and ionic complexes assigned as nCov(t), wCov, and Cov(pol) are expected to feature DE values in the range 11.27–12.24, 15.14–31.67, and 31.46–65.47 kcal mol^−1^, respectively. More precise estimates of the DEs could also be obtained by using Equations ((1), (2), (3)). To further explore the GLED assignment as a predictive tool of stability, and to eventually arrive at a classification of the HB, we performed the GLED analysis of other exemplary species, taken, in particular, from the NCI Atlas data set [75, 76]. The obtained results are discussed in the next subsection.

### 3.3 Hydrogen‐Bonded Systems From the NCI Atlas Data Set: A Proposed Classification of the HB

We investigated two groups of exemplary species taken from the NCI data set [75, 76]. The first one includes the 20 neutral complexes **1**–**20** listed in the first column of Table 4 and shown in Figure S3. These systems were selected so to sample species (i) stabilized by a single HB (ii) formed by ligands also different from those included in the ELKE data set and (iii) featuring DE values arriving up to more than 10 kcal mol^−1^. The results of the GLED analysis are given in Table 4. Complexes **1**–**10** were assigned as nCov(l), complex **11** was assigned as nCov(t), and complexes **12**–**20** were assigned as wCov. As shown in Table 4, the CCSD(T)/CBS DEs of complexes **1**–**6** fall in the range of 0.6–3.56 kcal mol^−1^ expected from the analysis of the ELKE data set for neutral species assigned as nCov(l). The DEs of complexes **7**–**10**, spanning between 3.63 and 4.35 kcal mol^−1^, are larger than the upper limit of 3.56 kcal mol^−1^, but by less than 1 kcal mol^−1^. The CCSD(T)/CBS DEs of these 10 species are also reasonably well predicted by Equation (1), with differences within 1 kcal mol^−1^, and a MAPE of 18.93%. The DE of complex **11** falls within the range of 3.73–5.43 kcal mol^−1^ expected from the analysis of the ELKE data set for neutral species assigned as nCov(t), and its CCSD(T)/CBS value of 4.46 kcal mol^−1^ is quite well reproduced by Equation (1). The CCSD(T)/CBS DEs of complexes **12**–**20** are, invariably, within the range of 4.93–13.36 kcal mol^−1^ expected from the analysis of the ELKE data set for species assigned as wCov, and their values are well reproduced by Equation (2), with a MAPE of 8.22%. A second group of investigated NCI Atlas species includes the 10 ionic complexes **1I**—**10I** listed in the first column of Table 5 and shown in Figure S4. They were selected so to sample species (i) stabilized by a single HB (ii) formed by cations (H_3_O^+^ and NH_4_^+^) and anions (CN^−^, OH^−^) also different from those included in the ELKE data set and (iii) featuring DE values arriving up to more than 40 kcal mol^−1^. The results of the GLED analysis are given in Table 5. Complexes **1I** and **2I** were assigned as nCov(t). Their DEs of 8.70 and 9.01 kcal mol^−1^, respectively, are quite well reproduced by Equation (3), but they are *ca*. 2 kcal mol^−1^ outside the range of 11.27–12.24 kcal mol^−1^ expected from the analysis of the ELKE data set for ionic species assigned as nCov(t). Complexes **3I**—**8I** were assigned as wCov, and their CCSD(T)/CBS DEs are, indeed, within the range of 15.14–31.67 kcal mol^−1^ expected from the analysis of the ELKE data set for ionic species assigned as wCov. The absolute values span between 16.58 and 31.70 kcal mol^−1^, and are relatively well reproduced by Equation (3), with a MAPE of 21.45%.

**TABLE 4:** GLED assignment, dissociation energy DE (kcal mol^−1^), electron density *ρ* (*e a*_0_^−3^), and energy density *H* (*hartree a*_0_^−3^) of the neutral hydrogen‐bonded complexes taken from the NCI Atlas data sets [75, 76] (see Figure S3).

Complex			DE					
Number—A/B pair	Contact	GLED assignment^a^	ELKE range^b^	CCSD(T)/CBS^c^	Calc.^d^	Δ^e^ (APE^f^)	*ρ*(BCP)^g^	*H*(BCP)^g^
**1**—H_2_O/CH_4_	O–H—C	nCov(l)	0.6–3.56	0.97	1.04	0.07 (6.93)	0.0074	0.0012
**2**—PH_3_/NH_3_	P–H—N	nCov(l)	0.6–3.56	1.08	1.28	0.20 (18.25)	0.0086	0.0009
**3**—C_2_H_2_/PH_3_	C–H—P	nCov(l)	0.6–3.56	1.44	1.08	−0.36 (25.19)	0.0076	0.0008
**4**—PH_3_/MeNH_2_	P–H—N	nCov(l)	0.6–3.56	1.71	1.32	−0.39 (22.98)	0.0088	0.0010
**5**—PH_3_/Me_3_N	P–H—N	nCov(l)	0.6–3.56	2.14	1.90	−0.24 (11.36)	0.0117	0.0011
**6**—C_2_H_2_/H_2_O	C–H—O	nCov(l)	0.6–3.56	2.85	2.50	−0.35 (12.40)	0.0147	0.0020
**7**—C_2_H_2_/NH_3_	C–H—N	nCov(l)	0.6–3.56	3.63	2.86	−0.77 (21.31)	0.0165	0.0010
**8**—C_2_H_2_/MeNH_2_	C–H—N	nCov(l)	0.6–3.56	4.15	3.40	−0.75 (18.17)	0.0192	0.0006
**9**—C_2_H_2_/Pyridine	C–H—N	nCov(l)	0.6–3.56	4.15	3.32	−0.83 (20.09)	0.0188	0.0009
**10**—NH_3_/Pyridine	N–H—N	nCov(l)	0.6–3.56	4.36	2.94	−1.42 (32.65)	0.0169	0.0012
**11**—H_2_S/Pyridine	S–H—N	nCov(t)	3.73–5.43	4.46	4.58	0.12 (2.59)	0.0251	−0.0006
**12**—MeOH/H_2_O	O–H—O	wCov (0.075)	4.93–13.36	5.08	5.02	−0.06 (1.09)	0.0266	−0.0016
**13**—HCl/H_2_O	Cl–H—O	wCov (0.116)	4.93–13.36	5.57	6.75	1.18 (21.11)	0.0337	−0.0040
**14**—H_2_O/MeOH	O–H—O	wCov (0.078)	4.93–13.36	5.76	5.70	−0.06 (0.98)	0.0294	−0.0027
**15**—MeOH/MeOH	O–H—O	wCov (0.078)	4.93–13.36	5.87	5.82	−0.05 (0.77)	0.0299	−0.0028
**16**—H_2_O/Pyridine	O–H—N	wCov (0.099)	4.93–13.36	7.08	6.75	−0.33 (4.72)	0.0337	−0.0048
**17**—H_2_O/MeNH_2_	O–H—N	wCov (0.103)	4.93–13.36	7.50	6.89	−0.61 (8.11)	0.0343	−0.0051
**18**—MeOH/Pyridine	O–H—N	wCov (0.098)	4.93–13.36	7.51	7.09	−0.42 (5.65)	0.0351	−0.0053
**19—**MeOH/MeNH_2_	O–H—N	wCov (0.105)	4.93–13.36	7.74	7.18	−0.56 (7.21)	0.0355	−0.0057
**20—**HCl/NH_3_	Cl–H—N	wCov (0.197)	4.93–13.36	9.68	12.03	2.35 (24.29)	0.0555	−0.0161

^a^ B3LYP‐D3(BJ)/ma‐TZVPP calculations at the B3LYP‐D3/def2‐QZVP optimized geometries taken from the references [75, 76]. For the HBs of covalent character, the value in parenthesis is the B3LYP‐D3(BJ)/ma‐TZVPP AIM delocalization index.

^b^ Values expected from the GLED analysis of the ELKE data set [69].

^c^ CCSD(T)/CBS dissociation energies calculated at the B3LYP‐D3/def2‐QZVP optimized geometries taken from the references [75, 76].

^d^ Calculated by Equation (1) for nCov(l)/nCov(t) or by Equation (2) for wCov.

^e^ Δ = DE (Calc.) – DE [CCSD(T)/CBS].

^f^ Absolute Percentage Error = |Δ |/DE[CCSD(T)/CBS] × 100.

^g^ Calculated at the B3LYP‐D3(BJ)/ma‐TZVPP AIM BCP.

**TABLE 5:** GLED assignment, dissociation energy DE (kcal mol^−1^), electron density *ρ* (*e a*_0_^−3^), and energy density *H* (*hartree a*_0_^−3^) of the ionic hydrogen‐bonded complexes taken from the NCI Atlas data sets [75, 76] (see Figure S4).

Complex	Contact	GLED assignment^a^	DE				*ρ*(BCP)^g^	*H*(BCP)^g^
Number—A/B pair			ELKE range^b^	CCSD(T)/CBS^c^	Calc.^d^	Δ^e^ (APE^f^)		
**1I**—NH_3_/CN^−^	N–H—C	nCov(t)	11.27–12.24	8.70	8.51	−0.19 (2.21)	0.0224	−0.0002
**2I**—NH_3_/NC^−^	N–H—N	nCov(t)	11.27–12.24	9.01	9.48	0.47 (5.21)	0.0253	−0.0005
**3I**—H_2_O/NC^−^	O–H—N	wCov (0.120)	15.14–31.67	16.58	16.58	−1.10 (6.65)	0.0432	−0.0089
**4I**—H_2_O/CN^−^	O–H—C	wCov (0.140)	15.14–31.67	16.67	14.41	−2.26 (13.59)	0.0400	−0.0079
**5I**—MeOH/CN^−^	O–H—C	wCov (0.150)	15.14–31.67	18.90	16.21	−2.69 (14.21)	0.0454	−0.0108
**6I**—H_3_O^+^/MeOCN	O–H—O	wCov (0.169)	15.14–31.67	22.42	33.57	11.15 (49.74)	0.0972	−0.0519
**7I**—NH_4_^+^/MeC(O)H	N–H—O	wCov (0.154)	15.14–31.67	26.21	22.72	−3.49 (13.33)	0.0648	−0.0204
**8I**—NH_4_^+^/MeOCN	N–H—N	wCov (0.163)	15.14–31.67	31.70	21.81	−9.89 (31.20)	0.0621	−0.0190
**9I**—MeC(O)OH/NC^−^	O–H—N	Cov(pol)(0.206)	31.46–65.47	30.75	31.46	0.71 (2.31)	0.0909	−0.0442
**10I**—H_2_O/OH^−^	O–H—O	Cov(pol)(0.272)	31.46–65.47	40.93	44.56	3.63 (8.87)	0.1300	−0.0924

^a^ B3LYP‐D3(BJ)/ma‐TZVPP calculations at the B3LYP‐D3/def2‐QZVP optimized geometries taken from the references [75, 76]. For the HBs of covalent character, the value in parenthesis is the B3LYP‐D3(BJ)/ma‐TZVPP AIM delocalization index.

^b^ Values expected from the GLED analysis of the ELKE data set [69].

^c^ CCSD(T)/CBS dissociation energies calculated at the B3LYP‐D3/def2‐QZVP optimized geometries taken from the references [75, 76].

^d^ Calculated by Equation (3).

^e^ Δ = DE (Calc.) – DE [CCSD(T)/CBS].

^f^ Absolute Percentage Error = |Δ |/DE[CCSD(T)/CBS] × 100.

^g^ Calculated at the B3LYP‐D3(BJ)/ma‐TZVPP AIM BCP.

Finally, the CCSD(T)/CBS values of the DEs of **9I** and **10I**, assigned as Cov(pol), fall within the range of 31.46–65.47 kcal mol^−1^ expected from the analysis of the ELKE data set for ionic species assigned as Cov(pol), and their absolute values are well reproduced by Equation (3).

Overall, the joint analysis of the ELKE [69] and of some exemplary NCI Atlas species [75, 76] confirms the strict relationship between the stability of the hydrogen‐bonded complexes and their GLED assignment. Noting, in particular, that the DEs increase by increasing the degree of covalency, we classify the nCov(l), nCov(t), wCov, and Cov(pol) interactions as weak, medium, strong, and very strong, respectively. These assignments are not inconsistent with former studies [97, 98] where classifications of the HB were based on energetic and AIM parameters. For example, the bonds occurring in complexes of ylides containing N, O, and C as HB acceptors [97] were classified as weak for values of *H*_BCP_ > 0 and DE < 12.0 kcal mol^−1^, and medium or strong for values of *H*_BCP_ < 0 and DE in the range 12.0–24 and > 24 kcal mol^−1^, respectively.

Based on the data quoted in Tables 4 and 5, any GLED category/strength labeling corresponds to a well defined range of DE values, and this produces the HB classification summarized in Table 6. The interactions occurring in neutral complexes are, in particular, assigned as weak, medium, and strong, and those occurring in ionic complexes are assigned as medium, strong, and very strong HB. For the various categories, more precise values of DE are expectedly obtained from the linear equations quoted as well in Table 6.

**TABLE 6:** Classification of the HB according to the GLED assignment of the bond.

Type of complex	GLED assignment	Classification	DE (kcal mol^−1^)	
			Expected range	Predicted value
Neutral	nCov(l)	Weak	0.5–4.5	DE = 199.90 × *ρ*(BCP) − 0.442
	nCov(t)	Medium	3.5–5.5	
	wCov	Strong	4.5–15.0	DE = 242.46 × *ρ*(BCP) − 1.425
Ionic	nCov(t)	Medium	8.5–13.0	DE = 335.08 × *ρ*(BCP) + 1.002
	wCov	Strong	15.0–32.0	
	Cov(pol)	Very strong	30.0–70.0	

### 3.4 GLED Analysis of Systems Containing More Than One HB

**FIGURE 6:**
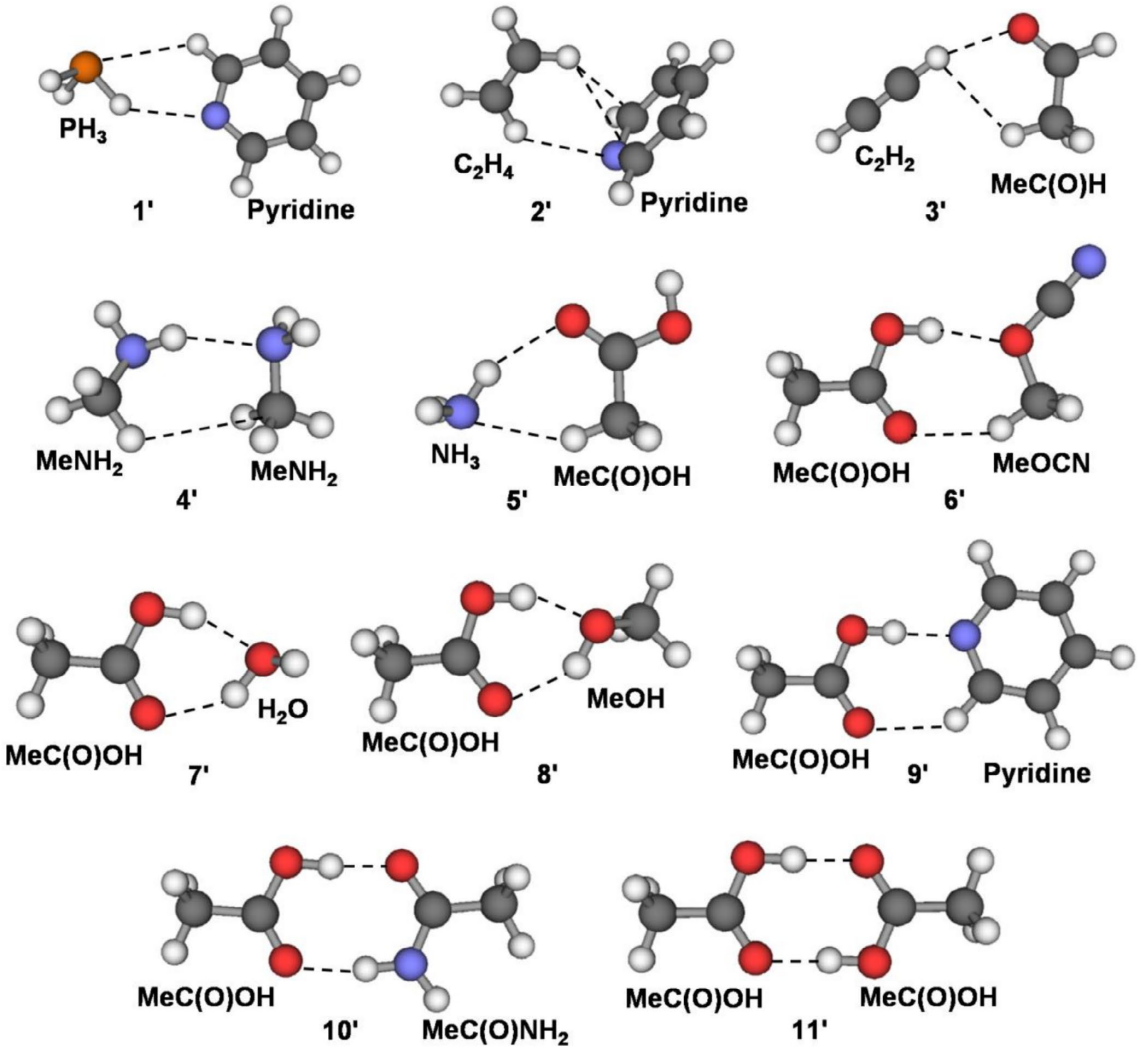
Neutral doubly coordinated hydrogen‐bonded complexes taken from the NCI Atlas data set [75, 76].

Numerous natural and synthetic processes involve molecular or supramolecular systems stabilized by two or more HBs. Thus, to appreciate the role of one or more specific contacts, it is necessary to disentangle the contribution of the various involved interactions. As discussed in this paragraph, useful insights in this regard could be obtained by our proposed GLED analysis and HB classification. We investigated, in particular, the 10 complexes **1′–10′** shown in Figure 6.

**FIGURE 7:**
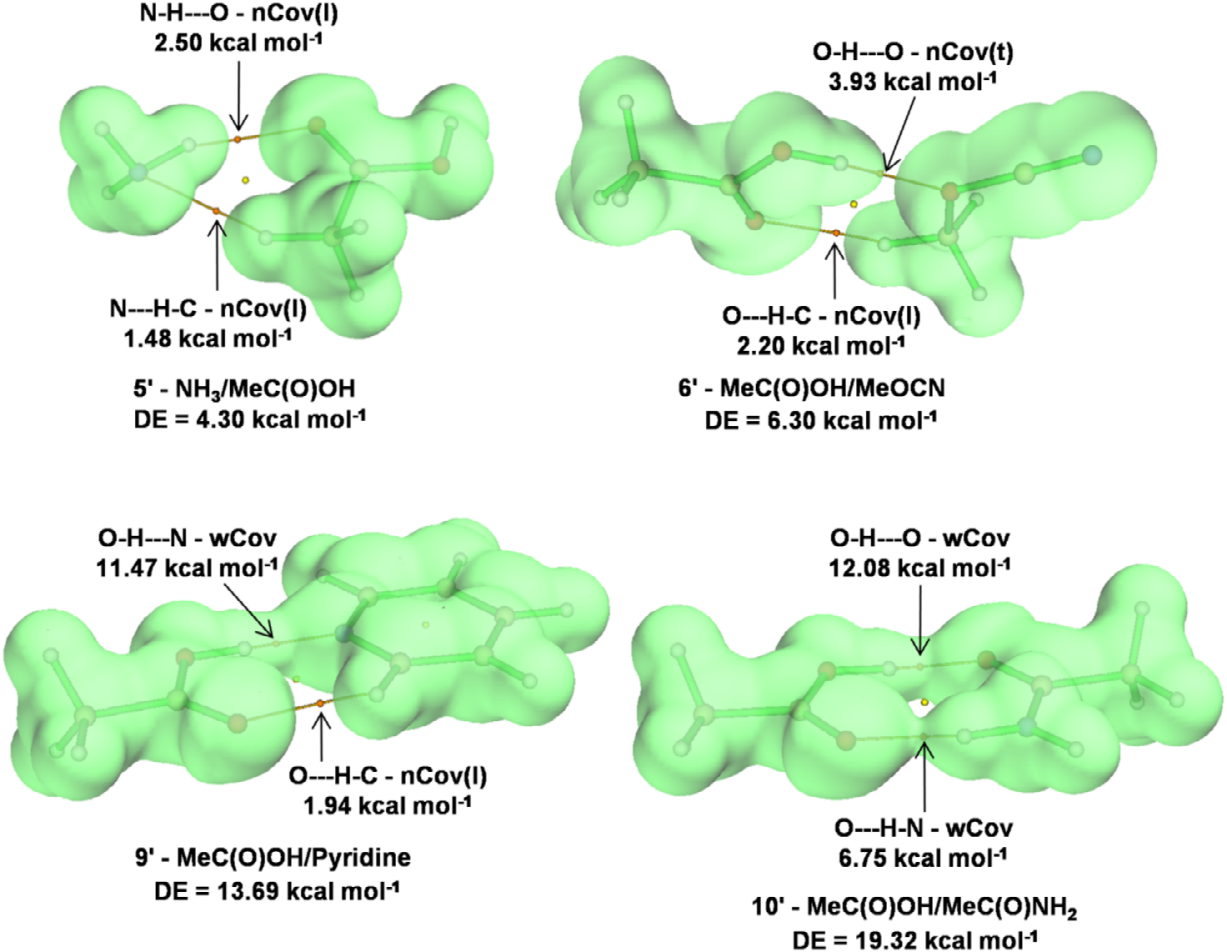
3D plot of the *H*(***r***) of some exemplary neutral doubly‐coordinated hydrogen‐bonded complexes taken from the NCI Atlas data set [75, 76]. The DE values are the total dissociation energies taken from the references [75, 76], and the contributions of the single interactions are calculated by Equations (1) or (2).

They were selected from the NCI Atlas data set so to (i) be stabilized by two HBs, (ii) cover values of DE up to *ca*. 20 kcal mol^−1^, and (iii) be of size small enough to ensure that stabilizing contributions other than the two HBs are negligible, if any. The results of the GLED analysis, and the quantitative data related with the HB classification, are given in Table 7, where the complexes are ranked by increasing values of DE. The occurring bonding situations are, essentially, four, namely two contacts both assigned as nCov(l), two contacts both assigned as wCov, and two contacts of different character assigned as nCov(t)/nCov(l), and wCov/nCov(l), respectively. The plotted *H*(0,ISO) of these various situations look like those shown in Figure 7 for the exemplary complexes **5′**, **6′**, **9′**, and **10′**. In complex **5′**, the *H*(0,ISO) of the NH_3_ and MeC(O)OH composing fragments are separated, and the *H*(***r***) is positive at the BCPs occurring on both the N–H—O and N—HC bonds. The interactions are, therefore, both assigned as nCov(l), and the values of *ρ*(BCP) furnish through Equation (1) DE values of 2.50 and 1.48 kcal mol^−1^, respectively. The sum of these values well reproduces the CCSD(T)/CBS DE of the complex of 4.30 kcal mol^−1^. The bonding situation of complexes **1′**–**4′** is strictly similar, featuring two or three nCov(l) contacts of clearly different estimated DEs (see Table 7), whose summed values again well reproduce the overall stability.

**TABLE 7:** GLED assignment, dissociation energy DE (kcal mol^−1^), electron density *ρ* (*e a*_0_^−3^), and energy density *H* (*hartree a*_0_^−3^) of the neutral hydrogen‐bonded complexes taken from the NCI Atlas data sets [75, 76] (see Figure 6).

Complex			DE					
Number—A/B pair	Contact	GLED assignment^a^	Expected range^b^	CCSD(T)/CBS^c^	Calc.^d^	Δ^e^ (APE^f^)	*ρ*(BCP)^g^	*H*(bcp)^g^
**1′**—PH_3_/Pyridine	P–H—N P—H–C	nCov(l) nCov(l)	0.5–4.5 0.5–4.5	1.92	1.30 0.51	−0.11 (5.73)	0.0087 0.0048	0.0011 0.0009
**2′**—C_2_H_4_/Pyridine	C–H—N C–H—C C–H—C	nCov(l) nCov(l) nCov(l)	0.5–4.5 0.5–4.5 0.5–4.5	2.57	0.99 0.42 0.42	−0.74 (28.9)	0.0072 0.0043 0.0043	0.0011 0.0009 0.0009
**3′**—C_2_H_2_/MeC(O)H	C–H—O C—H–C	nCov(l) nCov(l)	0.5–4.5 0.5–4.5	3.35	2.49 0.42	−0.44 (13.13)	0.0147 0.0043	0.0019 0.0009
**4′**—MeNH_2_/MeNH_2_	N–H—N C–H—C	nCov(l) nCov(l)	0.5–4.5 0.5–4.5	4.18	3.14 0.11	−0.93 (22.25)	0.0179 0.0028	0.0009 0.0007
**5′**—NH_3_/MeC(O)OH	N–H—O N—H–C	nCov(l) nCov(l)	0.5–4.5 0.5–4.5	4.30	2.50 1.47	−0.33 (7.67)	0.0147 0.0096	0.0020 0.0014
**6′**—MeC(O)OH/MeOCN	O–H—O O—H–C	nCov(t) nCov(l)	3.5–5.5 0.5–4,5	6.30	3.93 2.20	−0.17 (2.70)	0.0219 0.0132	−0.0002 0.0021
**7′**—MeC(O)OH/H_2_O	O–H—O O—H–O	wCov (0.096) wCov (0.069)	4.5–15.0 4.5–15.0	11.15	7.71 5.44	2.00 (17.94)	0.0377 0.0283	−0.0060 −0.0008
**8′**—MeC(O)OH/MeOH	O–H—O O—H–O	wCov (0.102) wCov (0.068)	4.5–15.0 4.5–15.0	12.01	8.90 5.32	2.21 (18.40)	0.0426 0.0278	−0.0088 −0.0005
**9′**—MeC(O)OH/Pyridine	O–H—N O—H–C	wCov (0.133) nCov(l)	4.5–15.0 0.5–4.5	13.69	11.47 1.94	−0.28 (2.05)	0.0532 0.0119	−0.0155 0.0020
**10′**—MeC(O)OH/MeC(O)NH_2_	O–H—O O—H–N	wCov (0.126) wCov (0.096)	4.5–15.0 4.5–15.0	19.32	12.08 6.75	−0.49 (2.54)	0.0557 0.0337	−0.0165 −0.0031
**11′**—MeC(O)OH/MeC(O)OH	O–H—O O—H–O	wCov (0.119) wCov (0.119)	4.5–15.0 4.5–15.0	20.52	11.52 11.52	2.52 (12.28)	0.0534 0.0534	−0.0151 −0.0151

^a^ B3LYP‐D3(BJ)/ma‐TZVPP calculations at the B3LYP‐D3/def2‐QZVP optimized geometries taken from the references [75, 76]. For the HBs of covalent character, the value in parentheses is the B3LYP‐D3(BJ)/ma‐TZVPP AIM delocalization index.

^b^ Values expected from the HB classification given in Table 6.

^c^ CCSD(T)/CBS dissociation energies calculated at the B3LYP‐D3/def2‐QZVP optimized geometries taken from the references [75, 76].

^d^ Calculated by Equation (1) for nCov(l)/nCov(t) or by Equation (2) for wCov.

^e^ Δ = DE (Calc.) − DE [CCSD(T)/CBS].

^f^ Absolute percentage error = |Δ |/DE[CCSD(T)/CBS] × 100.

^g^ Calculated at the B3LYP‐D3(BJ)/ma‐TZVPP AIM BCP.

In complex **6′**, the MeC(O)OH and MeOCN fragments are, again, separated, and the *H*(BCP) is positive at the O—HC bond, but negative at the O–H—O one. The interactions are, therefore, assigned as nCov(l) and nCov(t), respectively, and their stabilizing contributions are estimated by Equation (1) as 2.20 and 3.93 kcal mol^−1^, respectively. These values nearly exactly disentangle the CCSD(T)/CBS DE of 6.30 kcal mol^−1^.

In complex **9′**, the interaction between MeC(O)OH and Pyridine produces the overlapping of the *H*(0,ISO) of the fragments along the direction of the O–H—N contact, which is assigned as wCov based on the value of 0.133*e* of the *δ*(H,N). This interaction is predicted by Equation (2) to stabilize the complex by 11.47 kcal mol^−1^, which accounts for the by far greatest fraction of the CCSD(T)/CBS DE of 13.69 kcal mol^−1^. The residual contribution of *ca*. 2 kcal mol^−1^ is, therefore, referred to the O—H–C contact, whose GLED assignment as nCov(l) corresponds, indeed, to an estimated DE of 1.94 kcal mol^−1^. Finally, in complex **10′**, the interaction between MeC(O)OH and MeC(O)NH_2_ produces the overlapping of their *H*(0,ISO) along both the O–H—O and the O—H–N contact, which are assigned as wCov. The former has, however, a higher value of *ρ*(BCP) of (0.0557 vs. 0.0337*e a*_0_^−3^) and, in fact, Equation (2) partitions the CCSD(T)/CBS DE of 19.32 kcal mol^−1^ into appreciably different contributions of 12.08 (O–H—O) and 6.75 kcal mol^−1^ (O—H–N). Complexes **7′**, **8′**, and **11′** are as well stabilized by two wCov contacts, whose strength is, again, well predicted using the *ρ*(BCP) of the various occurring O–H—O contacts. The summed contributions furnish, in fact, a reasonably good estimate of the corresponding CCSD(T)/CBS DEs (see Table 7).

## 4 Concluding Remarks

Despite its formal simplicity, the HB is quite complex in nature, including binding components of different character and strength. Depending on the involved donor and acceptor, the interaction may range from a dispersive and electrostatic contact to a bond involving polarization and charge transfer. This change in bonding character typically mirrors an increase of the strength of the HB, which changes from a weak noncovalent contact to a strong covalent bond. The crossover of these components was demonstrated, for example, in a recent experimental study on the [F–H–F]^−^ [99, 100], and is also effectively caught by different techniques of bonding analysis. Consistent with these findings, the application of our recently proposed GLED method [71] to a group of exemplary hydrogen‐bonded structures actually confirmed the gradually varying nature of the HB, and allowed also to propose a classification based on the correlation between the assigned bonding character and the strength of the interaction. A distinct advantage of our approach is that it allows a direct visualization of the nature of the bond (noncovalent or with contribution of covalency), and, hence, of its strength, being informative in this regard the 3D representation of the *H*(***r***) = 0 isosurface. This is especially useful for systems stabilized by two or more HBs, whose in case different role can be eye‐caught by looking at the graph of the *H*(0,ISO). The GLED analysis is also of relatively low computational cost, thus allowing the investigation of large‐size species, including systems of biological interest. It is also in principle applicable to any type of intermolecular interaction [101, 102, 103, 104, 105], and we are planning future work aimed at their investigation and classification using an approach similar to the one adopted in the present study.

## Funding

This work was supported by the Rome Technopole Foundation, PNRR action in the field of the NextGenerationEU—Section 4.

## Conflicts of Interest

The authors declare no conflicts of interest.

## Supporting information


**Data S1:** jcc70348‐sup‐0001‐Supinfo.docx.

## Data Availability

The data that support the findings of this study are available from the corresponding author upon reasonable request.
