# Supplementary material for: A Theoretical Investigation on the Hydrogen Bond Based on the GLED Method of Bonding Analysis
Source: J Comput Chem. 2026 Mar 9;47(7):e70348. doi: 10.1002/jcc.70348 (PMC12969774; doi:10.1002/jcc.70348)
Supplement: Supplementary file 1 — Data S1: jcc70348‐sup‐0001‐Supinfo.docx. [file JCC-47-0-s001.docx]

*Supporting Information for*

**A Theoretical Investigation on the Hydrogen Bond Based on the GLED Method of Bonding Analysis**

Stefano Borocci,^1,2^ Felice Grandinetti,^1,2^ Nico Sanna,^1,3^ Costantino Zazza^1^

1. *Dipartimento per la Innovazione nei sistemi Biologici, Agroalimentari e Forestali (DIBAF), Università della Tuscia, L.go dell’Università, s.n.c., 01100 Viterbo (Italy)*
2. *Istituto per i Sistemi Biologici del CNR, Via Salaria, Km 29.500, 00015, Monterotondo, RM (Italy)*
3. *Istituto per la Scienza e Tecnologia dei Plasmi (ISTP) del CNR,Via Amendola, 122/D, 70126 Bari (Italy)*

**Correspondence**: Felice Grandinetti (fgrandi@unitus.it)

**DATA S1.** B3LYP-D3(BJ)/ma-TZVPP cartesian coordinates (Å) of the neutral and ionic hydrogen-bonded complexes included in the ELKE data set taken from the reference [70]. The total charge (*e*) is given in parenthesis.

**H_3_CH---NCH** (0)

C -2.65287800 0.00025600 0.00000300

H -3.01862900 1.02586900 -0.00082700

H -1.56508800 -0.00012800 -0.00007500

H -3.01865000 -0.51182000 0.88866700

H -3.01878900 -0.51330700 -0.88774500

N 1.24647300 -0.00110800 -0.00001600

C 2.39241300 0.00058000 0.00000600

H 3.45863700 0.00212800 0.00004100

**H_3_CH---OH_2_** (0)

C -1.89891700 -0.00014100 0.00709800

H -2.22446800 -0.36550600 0.98002700

H -0.81179900 -0.00319400 -0.04174000

H -2.30183900 -0.64472100 -0.77255600

H -2.26944300 1.01349200 -0.13844100

O 1.80988500 -0.00027800 -0.08106400

H 2.26359300 -0.76198600 0.28980200

H 2.25837600 0.76498500 0.28882700

**MeCCH---OC** (0)

C 1.92253200 -0.00178700 0.00022800

C 0.72272200 -0.00794000 0.00066600

C 3.37667800 0.00559400 -0.00033800

H -0.33875800 -0.01327700 0.00101000

H 3.76346000 0.58978100 -0.83678400

H 3.76491100 0.44071000 0.92186800

H 3.77183400 -1.00778300 -0.08657100

O -2.88163600 -0.00876200 -0.00053600

C -4.00665700 0.01424500 0.00023700

**H_3_CH---NH_3_** (0)

C -1.96643100 0.00002100 0.00044100

H -2.32855700 0.26392300 -0.99232200

H -0.87767100 0.00042000 0.00671700

H -2.33862800 0.72577700 0.72229500

H -2.33532700 -0.99007100 0.26496200

N 1.85650600 0.00005700 0.00136900

H 2.22675200 0.93689700 -0.10445100

H 2.21507000 -0.55519200 -0.76615000

H 2.24140100 -0.38227300 0.85671900

**HCCH---OC** (0)

C 0.06823600 2.92328500 0.00000000

H 0.10033200 3.98475600 0.00000000

C 0.03197900 1.72675500 0.00000000

H 0.00000000 0.66485900 0.00000000

O -0.04997400 -1.84264500 0.00000000

C -0.05030600 -2.96811600 0.00000000

**FCCH---OC** (0)

C -0.12597000 -1.97614600 0.00000000

C -0.05960100 -0.78545300 0.00000000

F -0.19748200 -3.25219900 0.00000000

H 0.00000000 0.27357000 0.00000000

O 0.16546300 2.77409400 0.00000000

C 0.26117700 3.89551000 0.00000000

**HOH---OC** (0)

O -2.14959900 -0.15587200 -0.00438700

H -1.24432200 0.16623600 -0.02624300

H -2.69604100 0.62975700 0.07860200

O 1.05980600 0.20563300 -0.01669500

C 2.10978500 -0.19901300 0.01938300

**HSH---SH_2_** (0)

S -2.06785100 -0.08615900 0.00000400

H -0.73058500 0.06887200 -0.00044300

H -2.28437900 1.23773000 -0.00005600

H 2.06835200 -0.81679900 0.97101400

H 2.06964000 -0.81776100 -0.97006800

S 1.99766200 0.10665700 -0.00003200

**HCCH---SH_2_** (0)

C -3.11657100 0.00029800 0.04314200

H -4.17399800 0.00054100 0.14040900

C -1.92414500 -0.00052500 -0.06869500

H -0.86274500 -0.00103600 -0.16429700

H 2.02141300 -0.96818600 0.83390100

H 2.01832900 0.97190500 0.82997700

S 1.95258100 -0.00011700 -0.09291700

**HSH---N_3_H** (0)

S 2.38559300 -0.15964600 -0.08496200

H 2.59340400 -0.07021100 1.23740900

H 1.10999100 0.25520800 0.02196800

N -1.09378700 0.88654600 0.05787400

H -1.42803700 1.78700000 -0.27726900

N -1.98835100 0.03493800 -0.00372500

N -2.69569700 -0.83829200 -0.00025300

**HSH---OH_2_** (0)

S -1.33356600 -0.08644300 0.00013900

H 0.00896000 0.01569900 0.00224300

H -1.50145500 1.24432100 -0.00236400

H 2.66247800 -0.25980900 0.76417700

H 2.65552600 -0.26285000 -0.76692900

O 2.18894400 0.08071600 0.00008000

**HOH---SH_2_** (0)

O -2.22439500 0.11085800 -0.00369100

H -1.28540900 -0.11854300 -0.00090200

H -2.69007000 -0.72881900 0.01980000

S 1.21561900 -0.10487900 0.00615800

H 1.14419100 0.87086200 0.92493200

H 1.17654300 0.76770900 -1.01283100

**HCCH---OH_2_** (0)

C 2.24670600 -0.00112000 -0.01841000

H 3.30798100 -0.00479400 -0.05001800

C 1.04912700 0.00259700 0.02109200

H -0.01828300 0.00590900 0.05244500

O -2.20677400 0.00041800 0.05809500

H -2.69613500 -0.77168100 -0.23875100

H -2.71436700 0.75835600 -0.24452400

**N_3_H---FH** (0)

N 0.30150900 0.80685900 -0.00667100

H -0.57582000 0.28843400 -0.04260500

N 1.26914900 0.04497600 -0.00070500

N 2.23952100 -0.52671000 0.01007700

H -3.20849200 0.30112600 0.15316000

F -2.54299300 -0.31838200 -0.01438600

**H_2_NH---NH_3_** (0)

N -1.67894600 0.00000500 0.10223900

H -2.10781000 -0.81468900 -0.31959700

H -0.69331000 -0.00004900 -0.15080600

H -2.10772000 0.81472400 -0.31964100

N 1.56378900 0.00000500 -0.03552900

H 1.53945200 0.00015800 0.97741300

H 2.08785700 0.81612100 -0.32728700

H 2.08763500 -0.81633500 -0.32705800

**N_3_H---SH_2_** (0)

N -1.09676200 0.95941000 0.00025600

H -0.13982000 0.59221100 -0.00026400

N -1.91587100 0.04068200 0.00004600

N -2.76632700 -0.69865600 -0.00015300

H 2.58502900 0.60967300 -0.96707400

H 2.58343700 0.60030600 0.97481600

S 2.21400400 -0.24451600 -0.00053300

**HSH-NH_3_** (0)

S 1.34033700 -0.08093000 0.00000600

H -0.01283900 0.00281100 -0.00119300

H 1.49424300 1.25155000 -0.00003500

N -2.18223100 0.00830500 -0.00010800

H -2.53145000 -0.53142500 -0.78293800

H -2.58763200 0.93436400 -0.06344000

H -2.53209900 -0.42055000 0.84826100

**HCCH---NH_3_** (0)

C -2.28381500 0.00001700 -0.00020000

H -3.34555700 0.00019100 -0.00056900

C -1.08489900 -0.00006700 0.00043000

H -0.01241800 -0.00019600 0.00093200

N 2.24520400 -0.00000100 0.00007000

H 2.61686800 0.86096500 -0.38353500

H 2.61695000 -0.76183500 -0.55487400

H 2.62001000 -0.09881800 0.93617700

**HOH---N_3_H** (0)

O 2.55632000 -0.31451600 -0.12060000

H 2.96136400 -0.36132600 0.74872400

H 1.66417000 0.02686000 0.03136500

N -0.23619700 0.79492900 0.09491000

H -0.39488700 1.68754700 -0.36668500

N -1.23225800 0.06619500 -0.00405300

N -2.05743100 -0.69497400 -0.01208600

**FH---FH** (0)

F -1.42449500 0.02091700 0.00001200

H -0.50659000 -0.12955100 -0.00016500

F 1.29600200 -0.09086500 0.00000900

H 1.66303200 0.75908500 -0.00002200

**HOH---OH_2_** (0)

O 1.50999300 0.00004300 -0.12085200

H 0.55410200 -0.00020200 0.03711100

H 1.91258000 -0.00024200 0.75052600

O -1.38149500 -0.00004700 0.10829800

H -1.74757600 -0.76572900 -0.34388800

H -1.74708900 0.76620500 -0.34332100

**FH---SH_2_** (0)

F -2.08064600 0.00020200 0.01202800

H -1.14526600 0.00295700 -0.05924300

H 1.22616900 0.96687700 0.81712100

H 1.21218000 -0.97631800 0.80856200

S 1.08954600 0.00029200 -0.10466800

**N_3_H---OH_2_** (0)

N -0.32826000 0.91343000 -0.00400400

H 0.60695900 0.49142300 -0.00613500

N -1.20602300 0.05353700 -0.00033400

N -2.10417800 -0.62841700 0.00299600

H 2.93780600 -0.22006000 -0.76981400

H 2.94166500 -0.15794600 0.76279200

O 2.37285000 -0.31040800 0.00281900

**HOH---NH_3_** (0)

O -0.03836200 1.54613400 0.00000000

H 0.06356500 0.57566500 0.00000000

H 0.85354600 1.90026300 0.00000000

N -0.03836200 -1.37627100 0.00000000

H -1.03728700 -1.54652400 0.00000000

H 0.34780000 -1.83229000 0.81779500

H 0.34780000 -1.83229000 -0.81779500

**FH---N_3_H** (0)

H -1.52874300 0.03664100 0.00019200

F -2.38985500 -0.34300500 -0.00006800

N 0.10445200 0.75452700 0.00033100

H 0.31346600 1.74935500 -0.00121600

N 1.14302800 0.07764200 -0.00005400

N 1.99880000 -0.64630500 -0.00004300

**N_3_H---NH_3_** (0)

N 0.33297100 0.91171000 -0.00003500

H -0.61247900 0.48097100 0.00101700

N 1.21470700 0.06125800 0.00006600

N 2.11008500 -0.62784100 -0.00001600

N -2.39388700 -0.25422000 0.00019900

H -3.04298800 0.51044400 -0.14491200

H -2.63769400 -0.69632700 0.87872600

H -2.55397100 -0.93143000 -0.73632900

**FH---OH_2_** (0)

F 1.40367200 0.00006400 0.01458900

H 0.46285900 0.00043000 -0.05057500

O -1.22862400 0.00019400 -0.09039500

H -1.63131800 -0.77063200 0.32058100

H -1.63559500 0.76807100 0.32184900

**FH---NH_3_** (0)

F -1.41764700 -0.00003700 0.00013900

H -0.45505600 -0.00052000 0.00174500

N 1.21213100 -0.00010700 0.00038300

H 1.57444700 0.94517700 -0.03976800

H 1.57227300 -0.50459200 -0.80102700

H 1.58224600 -0.43898500 0.83511700

**[H_2_SH---FH]^+^** (+1)

S 1.14057900 0.00027800 -0.10375600

H -0.23171000 0.00132000 -0.17963000

H 1.19588100 0.98914600 0.81971800

H 1.19374400 -0.99429800 0.81370700

F -1.95833300 -0.00032900 0.06395000

H -2.78218500 0.00234500 -0.36925300

**[H_3_NH---FH]^+^** (+1)

N -1.35657200 0.00892900 0.00006400

H -1.60597800 -0.77161400 -0.61118500

H -1.80633200 0.86141200 -0.33939600

H -1.67961600 -0.18976900 0.94923700

H -0.32937200 0.12692000 0.00154300

F 1.40476500 -0.04192100 -0.00029700

H 2.27441600 0.28783800 0.00203100

**[HOH---SH]^-^** (-1)

O -2.05796500 -0.01030200 -0.09132100

H -1.07157100 0.01119900 -0.22016500

H -2.12149200 0.01646200 0.86671600

S 1.14758800 0.08188000 0.00402600

H 1.29537900 -1.25532600 0.01959900

**[H_2_NH---OH]^-^** (-1)

N 1.34309100 0.00005300 -0.13723600

H 1.63477300 0.80744300 0.40725400

H 1.63517100 -0.80752500 0.40675600

H 0.27049800 -0.00016900 -0.09897700

O -1.38823600 -0.00006200 0.12198000

H -1.83618700 0.00037500 -0.73022500

**[H_2_NH---F]^-^** (-1)

N 1.28647000 -0.00000200 -0.12515700

H 1.55867900 -0.80649800 0.43029300

H 0.21821200 0.00029700 -0.12188900

H 1.55908400 0.80623900 0.43047400

F -1.37125200 -0.00000300 0.01524700

**[H_2_OH---FH]^+^** (+1)

O -1.22814600 0.00006700 0.09564200

H -0.20766800 0.00009300 0.06414900

H -1.62583300 -0.80628200 -0.28082800

H -1.62605800 0.80589400 -0.28169300

F 1.26062800 -0.00011500 -0.09100100

H 1.93907600 0.00079100 0.55224700

**[H_3_NH---OH_2_]^+^** (+1)

N -1.33605900 -0.00018800 0.00032200

H -1.68293900 -0.33859600 0.89878900

H -1.68716400 0.94548200 -0.15577500

H -0.27369900 0.00201900 0.00012300

H -1.68342800 -0.60759200 -0.74285300

O 1.35274900 0.00023100 -0.00156000

H 1.92729300 -0.77347600 0.00479600

H 1.93036200 0.77163100 0.00514400

**[H_3_NH---NH_3_]^+^** (+1)

N 0.00000000 0.00000000 -1.33650300

H 0.92231100 0.24592600 -1.69384000

H -0.67413400 0.67578200 -1.69384000

H -0.24817700 -0.92170800 -1.69384000

H 0.00000000 0.00000000 -0.19291800

N 0.00000000 0.00000000 1.34743600

H 0.66424300 -0.66660700 1.73263500

H -0.90942000 -0.24194800 1.73263500

H 0.24517700 0.90855400 1.73263500

**[HOH---F]^-^** (-1)

O 0.04769200 -1.20641300 0.00000000

H 0.08344700 -0.14599300 0.00000000

H -0.89421300 -1.38734100 0.00000000

F 0.04769200 1.24273700 0.00000000

**[HOH---OH]^-^** (-1)

O 1.23229200 -0.09785900 -0.05356300

H 0.11486500 -0.03008900 -0.07121500

H 1.48611700 0.65574500 0.48418400

H -1.55232300 -0.59996400 0.52266300

O -1.23837500 0.09464700 -0.06339100

**[H_2_OH---SH_2_]^+^** (+1)

O -1.81280000 -0.00106600 0.07747900

H -0.64215200 0.00703800 -0.01482700

H -2.25838800 -0.73139700 -0.38137500

H -2.26064600 0.83677300 -0.12004900

S 1.06777200 -0.09700900 -0.04482800

H 1.31395200 1.10561000 -0.59973300

H 1.26528800 0.34264900 1.21340900

**[Cl---H---Cl]^-^** (-1)

Cl 0.00000000 0.00000000 1.57343100

H 0.00000000 0.00000000 -0.01191900

Cl 0.00000000 0.00000000 -1.57273000

**[H_2_O---H---OH_2_]^+^** (+1)

O 1.19798400 -0.03521700 -0.05282500

H -0.00018600 0.02328600 -0.00011400

H 1.70003700 0.72820600 -0.37056300

H 1.66310500 -0.45795600 0.68327400

O -1.19794900 -0.03543100 0.05276000

H -1.66337600 -0.45558000 -0.68462700

H -1.69986000 0.72722900 0.37254800

**[F---H---F]^-^** (-1)

F 0.00000000 0.00000000 -1.14876900

H 0.00000000 0.00000000 -0.00016700

F 0.00000000 0.00000000 1.14878800

**DATA S2.** B3LYP-D3/def2-QZVP cartesian coordinates (Å) of the neutral and ionic hydrogen-bonded complexes formed by the A/B pairs taken from the NCI Atlas data sets (references [76] and [77]). The string is the NCI Atlas reference number, and the value in parenthesis is the total charge (*e*).

**Complex 1 - H_2_O/CH_4_ - HB375_7.104** (0)

C -1.846101358 0.005221557 0.000000000

H -1.398657259 -0.428951161 -0.891998789

H -2.911955847 -0.208682023 0.000000000

H -1.398657259 -0.428951161 0.891998789

H -1.696249032 1.082514894 0.000000000

O 1.686565461 -0.054941367 0.000000000

H 1.905958185 0.879676014 0.000000000

H 0.726394304 -0.085722688 0.000000000

**Complex 2 – PH_3_/NH_3_ - HB300SPX_1.01** (0)

P 1.201269535 -0.204173999 -0.693599141

H 0.007111355 -0.041005705 0.052498122

H 1.148472001 -1.620791091 -0.659628284

H 2.042957921 -0.122447272 0.444639236

N -2.317383177 0.467148893 1.239718272

H -3.069811028 0.670672488 0.594012941

H -2.592189466 -0.346791115 1.774918965

H -2.256951963 1.244567625 1.885050978

**Complex 3 – C_2_H_2_/PH_3_ - HB300SPX_3.02** (0)

C 0.000000265 2.043588826 0.000000000

H 0.000000179 0.979121358 0.000000000

C 0.000000282 3.239753475 0.000000000

H 0.000000279 4.300903661 0.000000000

P -0.000000232 -1.954011618 0.000000000

H -0.598123885 -2.711568021 1.035981141

H -0.598123885 -2.711568021 -1.035981141

H 1.196247927 -2.711567850 0.000000000

**Complex 4 - PH_3_/MeNH_2_ - HB300SPX_1.02** (0)

P 1.605548349 -0.390617824 -0.727405998

H 0.882634510 -0.288173574 0.486365489

H 2.871614111 -0.472756002 -0.095217766

H 1.688520054 1.012884015 -0.909617199

N -1.579614155 0.122719706 1.387937882

H -1.780112599 0.803508459 2.107918362

H -2.116964742 -0.705520914 1.605739010

C -1.927890779 0.640066284 0.064914757

H -1.685292782 -0.108488648 -0.688474052

H -2.980359683 0.917586721 -0.061457307

H -1.321017253 1.519654050 -0.147113262

**Complex 5 – PH_3_/Me_3_N - HB300SPX_1.04** (0)

P 0.084011746 -2.570366557 0.000000000

H 0.634622704 -1.263582160 0.000000000

H 0.930911127 -3.042279034 1.033886520

H 0.930911127 -3.042279034 -1.033886520

H 0.475218246 3.295382004 0.000000000

H 1.646111440 2.296365009 0.882652225

H 1.646111440 2.296365009 -0.882652225

H -0.066148445 1.123891618 -2.080724731

H -1.378505914 2.018668741 -1.289433942

H -1.319485420 0.248237817 -1.196974911

H -1.378505914 2.018668741 1.289433942

H -1.319485420 0.248237817 1.196974911

C 1.008685514 2.329911240 0.000000000

N 0.107770787 1.192825402 0.000000000

C -0.706536290 1.148871045 -1.199849959

C -0.706536290 1.148871045 1.199849959

H -0.066148445 1.123891618 2.080724731

**Complex 6 – C_2_H_2_/H_2_O - HB375_5.014** (0)

C -0.998102419 0.000000000 0.027389486

H 0.068468561 0.000000000 0.068057451

C -2.193935042 0.000000000 -0.021744174

H -3.254139240 0.000000000 -0.063600906

O 2.254394342 0.000000000 0.035184770

H 2.718719565 -0.764245022 -0.315113640

H 2.718719565 0.764245022 -0.315113640

**Complex 7 - C_2_H_2_/NH_3_ - HB375_6.002** (0)

C -0.980342327 -0.000000374 0.000000000

H 0.091450754 -0.000000299 0.000000000

C -2.177833273 0.000000216 0.000000000

H -3.238844962 0.000000910 0.000000000

N 2.347305947 0.000000188 0.000000000

H 2.720458963 -0.470564313 -0.815040377

H 2.720458963 -0.470564313 0.815040377

H 2.720460839 0.941127272 0.000000000

**Complex 8 - C_2_H_2_/MeNH_2_ - HB375_6.006** (0)

C -1.597952042 0.186011894 0.000000000

H -0.571514692 0.500643864 0.000000000

C -2.740858305 -0.171969589 0.000000000

H -3.754198053 -0.486617119 0.000000000

N 1.616743136 0.690093678 0.000000000

H 1.997761137 1.156502917 -0.812477095

H 1.997761137 1.156502917 0.812477095

C 1.968525879 -0.732595362 0.000000000

H 1.531924955 -1.208655425 0.876689260

H 3.043941315 -0.937201051 0.000000000

H 1.531924955 -1.208655425 -0.876689260

**Complex 9 - C_2_H_2_/Pyridine - HB375_6.009** (0)

C -3.259011169 -0.194006028 0.000000000

H -2.228267970 -0.488748998 0.000000000

C -4.406794317 0.147405679 0.000000000

H -5.425095934 0.445506402 0.000000000

H 1.717580494 2.426627915 0.000000000

H -0.604851808 1.544763183 0.000000000

C 2.591749646 0.455710896 0.000000000

C 1.540416747 1.361232517 0.000000000

C 0.241839253 0.868792167 0.000000000

N -0.045774092 -0.432624208 0.000000000

C 0.970195916 -1.295241609 0.000000000

C 2.301954732 -0.901502651 0.000000000

H 0.707071784 -2.345614444 0.000000000

H 3.087663086 -1.642585785 0.000000000

H 3.616137044 0.800402903 0.000000000

**Complex 10 - NH_3_/Pyridine - HB375_4.016** (0)

N -3.595113002 -0.074407035 0.000000000

H -2.719733327 0.443379929 0.000000000

H -4.122384425 0.215651098 0.813579457

H -4.122384425 0.215651098 -0.813579457

H 1.084823162 -2.439443966 0.000000000

H -1.185671976 -1.412994446 0.000000000

C 2.077453129 -0.526114331 0.000000000

C 0.972549819 -1.365158587 0.000000000

C -0.295301613 -0.796886929 0.000000000

N -0.502123717 0.521360976 0.000000000

C 0.565248108 1.319520413 0.000000000

C 1.870947569 0.846347942 0.000000000

H 0.367749601 2.384460772 0.000000000

H 2.700225478 1.538497855 0.000000000

H 3.079050123 -0.932583733 0.000000000

**Complex 11 - H_2_S/Pyridine - HB300SPX_1.13** (0)

H 1.250211536 1.596045702 0.136912607

H 3.150914906 1.841939977 0.530169019

S 2.023375364 2.538594674 0.732605162

H -1.865186527 -1.858400592 -2.416218182

H -0.326804170 0.091249994 -2.354035120

C -1.737905474 -2.176320753 -0.287359210

C -1.441305450 -1.529344141 -1.478925618

C -0.581395602 -0.439560237 -1.445313059

N -0.026963747 0.014389495 -0.321916209

C -0.316198520 -0.611341707 0.818509764

C -1.164320017 -1.708837131 0.886551519

H 0.151853765 -0.213582330 1.710630495

H -1.367046291 -2.180799665 1.836575038

H -2.403339684 -3.027877214 -0.274186504

**Complex 12 - MeOH/H_2_O - HB375_1.029** (0)

O -0.757723424 0.640840597 0.000000000

H 0.153912854 0.320518766 0.000000000

C -1.632556266 -0.471743904 0.000000000

H -2.650698917 -0.086847036 0.000000000

H -1.504208428 -1.099180090 0.887993138

H -1.504208428 -1.099180090 -0.887993138

O 2.019767964 -0.186804915 0.000000000

H 2.462995003 0.189477597 -0.765134271

H 2.462995003 0.189477597 0.765134271

**Complex 13 - HCl/H_2_O - HB300SPX_2.31** (0)

H -0.064352184 -0.188326900 -0.093549965

Cl 0.085737575 0.878306338 0.638965120

O -0.200326781 -1.677115184 -1.185430212

H -0.318639179 -1.442493303 -2.110104504

H 0.587456559 -2.227042661 -1.153100951

**Complex 14 - H_2_O/MeOH - HB375_1.052** (0)

O -2.011851377 0.172486548 -0.057464338

H -1.115176959 -0.185826244 0.033281139

H -2.491709977 -0.144630420 0.709936574

O 0.720765428 -0.645170790 0.053569011

H 0.951692672 -1.213076049 -0.685000266

C 1.512820807 0.543062246 -0.000695559

H 1.201144722 1.162982332 0.834994563

H 1.345728717 1.095970244 -0.926567928

H 2.575296703 0.316493286 0.103476198

**Complex 15 - MeOH/MeOH - HB375_1.023** (0)

O 1.314820958 -0.412457918 -0.489820465

H 0.450880825 0.014371030 -0.399320006

C 2.216978094 0.189483065 0.418542323

H 3.175611578 -0.315213487 0.312205478

H 1.889448906 0.084861602 1.458255496

H 2.366309090 1.253907523 0.209865811

O -1.317540329 0.626300044 -0.148976454

H -1.708256751 1.037926177 -0.922944424

C -2.142131387 -0.460577513 0.275791009

H -1.645605290 -0.912170756 1.129826507

H -2.247542785 -1.215206206 -0.505414034

H -3.129559808 -0.112485278 0.583646232

**Complex 16 - H_2_O/Pyridine - HB375_3.044** (0)

O -3.475931869 -0.000000063 -0.087855349

H -2.501930068 -0.000000050 -0.021587774

H -3.789910179 0.000001147 0.818290805

H 1.986689127 2.146980222 -0.026162115

H -0.496468268 2.052302040 0.037791171

C 2.185067759 0.000000007 -0.029310792

C 1.478484068 1.194262643 -0.010996002

C 0.091716418 1.143668238 0.025824530

N -0.592818099 -0.000000010 0.045272845

C 0.091716396 -1.143668253 0.025824563

C 1.478484064 -1.194262631 -0.010995977

H -0.496468255 -2.052302047 0.037791228

H 1.986689149 -2.146980202 -0.026162074

H 3.265429842 -0.000000006 -0.058657649

**Complex 17 - H_2_O/MeNH_2_ - HB375_3.040** (0)

O -1.952573200 0.183687112 0.000000000

H -1.074646913 -0.247298404 0.000000000

H -2.591802599 -0.530867844 0.000000000

N 0.782152108 -0.709179755 0.000000000

H 1.059489442 -1.243541127 0.812943926

H 1.059489442 -1.243541127 -0.812943926

C 1.423862569 0.612034627 0.000000000

H 1.094159054 1.165949844 -0.876570680

H 2.517090772 0.579606101 0.000000000

H 1.094159054 1.165949844 0.876570680

**Complex 18 - MeOH/Pyridine - HB375_3.022** (0)

O 2.918572453 0.000000000 -0.605637020

H 1.949094148 0.000000000 -0.504913136

C 3.497253484 0.000000000 0.681677575

H 4.579607695 0.000000000 0.560896664

H 3.221051514 0.887249147 1.263031668

H 3.221051514 -0.887249147 1.263031668

H -2.479468939 2.146999384 0.149245623

H -0.018739313 2.052345875 -0.190758575

C -2.675660888 0.000000000 0.177382161

C -1.975581823 1.194345616 0.080819669

C -0.601343049 1.143788024 -0.108281371

N 0.076852238 0.000000000 -0.200914078

C -0.601343049 -1.143788024 -0.108281371

C -1.975581823 -1.194345616 0.080819669

H -0.018739313 -2.052345875 -0.190758575

H -2.479468939 -2.146999384 0.149245623

H -3.746375272 0.000000000 0.324301814

**Complex 19 - MeOH/MeNH_2_ - HB375_3.018** (0)

O -1.381580817 0.540266585 -0.400640626

H -0.453659817 0.613664390 -0.105659031

C -1.981236071 -0.539769485 0.280588208

H -3.016523899 -0.611018642 -0.049777707

H -1.490933710 -1.495750111 0.060514036

H -1.982737432 -0.398740089 1.367695104

N 1.400011094 0.590126126 0.332033260

H 1.605487033 0.655972265 1.320229476

H 1.800631933 1.409079103 -0.106654200

C 1.960892139 -0.638396160 -0.242084504

H 1.693776595 -0.691501936 -1.295705972

H 3.048153765 -0.720136210 -0.155958378

H 1.513445069 -1.498924472 0.251950450

**Complex 20 - HCl/NH_3_ - HB300SPX_1.22** (0)

H 0.000000216 -0.312689967 0.000000000

Cl -0.000001282 1.030655196 0.000000000

N 0.000002744 -2.034483854 0.000000000

H -0.473181387 -2.393491906 0.819577485

H -0.473181387 -2.393491906 -0.819577485

H 0.946368920 -2.393493686 0.000000000

**Complex 1' – PH_3_/Pyridine - HB300SPX_1.03** (0)

P -0.073492991 -3.483349359 0.000000000

H 0.782083498 -2.355114525 0.000000000

H 0.628233930 -4.150964849 1.034438818

H 0.628233930 -4.150964849 -1.034438818

H -2.456404762 1.602310352 0.000000000

H -1.249575747 -0.566175388 0.000000000

C -0.633618250 2.753674197 0.000000000

C -1.376396919 1.581781693 0.000000000

C -0.700504509 0.367668002 0.000000000

N 0.628514058 0.269030062 0.000000000

C 1.332408858 1.400670544 0.000000000

C 0.751141680 2.662537124 0.000000000

H 2.410332061 1.293973718 0.000000000

H 1.371273501 3.547048557 0.000000000

H -1.123076565 3.717408329 0.000000000

**Complex 2' - C_2_H_4_/Pyridine - HB375_7.072** (0)

H -2.393608658 -1.079283453 -0.000000000

H -4.089394920 -0.348731589 -0.000000000

H -3.108512908 1.907742281 0.000000000

H -1.421114751 1.171193523 0.000000000

C -3.018073539 -0.196392641 -0.000000000

C -2.491589285 1.018755217 0.000000000

H 1.600339912 0.884179508 2.146607049

H 0.307353509 -1.230368114 2.052296941

C 1.709262578 1.053539913 0.000000000

C 1.339605724 0.448971585 1.192996603

C 0.616750464 -0.736440543 1.139544075

N 0.258234125 -1.326839712 0.000000000

C 0.616750464 -0.736440543 -1.139544075

C 1.339605724 0.448971585 -1.192996603

H 0.307353509 -1.230368114 -2.052296941

H 1.600339912 0.884179508 -2.146607049

H 2.270398009 1.977349128 0.000000000

**Complex 3' - C_2_H_2_/MeC(O)H - HB375_5.001** (0)

C -2.011548349 0.283130903 -0.000000131

H -1.057741761 0.762468607 -0.000000367

C -3.070459929 -0.274876730 0.000000058

H -4.013236394 -0.761855986 0.000000226

H 1.940821318 -1.758125761 0.875108408

H 1.940821279 -1.758126074 -0.875108172

H 0.427130498 -1.393607536 0.000000104

H 2.985734307 0.372943524 -0.000000415

C 1.506149660 -1.269746698 0.000000037

C 1.895294274 0.176459777 -0.000000126

O 1.121515189 1.100311531 0.000000134

**Complex 4' - MeNH_2_/MeNH_2_ - HB375_4.040** (0)

N 1.427339082 -0.599460933 -0.391210405

H 0.524716799 -0.814135861 0.022375840

H 2.004866126 -1.425237666 -0.309450334

C 2.047308300 0.531660202 0.293967970

H 3.009553628 0.759268640 -0.165146751

H 2.214250713 0.389080652 1.369340867

H 1.417609392 1.413212598 0.173110974

N -1.664130636 -0.604889635 0.320536536

H -2.004227267 -1.300880938 -0.329911309

H -2.236721794 -0.673068577 1.151113651

C -1.746377967 0.735799002 -0.266821839

H -1.398748668 1.466062795 0.462478458

H -2.749490135 1.031419200 -0.591364401

H -1.077169846 0.787266247 -1.123910106

**Complex 5' - NH_3_/MeC(O)OH - HB375_2.016** (0)

N -2.919078130 -0.005279467 0.000000000

H -2.102771897 0.598398113 0.000000000

H -3.473183159 0.230195710 0.813481220

H -3.473183159 0.230195710 -0.813481220

H 2.328249337 1.085668782 0.000000000

H -0.798340011 -1.399454574 0.000000000

H 0.681937940 -1.880705291 0.876460187

H 0.681937940 -1.880705291 -0.876460187

O 0.055462564 1.036618867 0.000000000

C 0.755787335 0.054599507 0.000000000

O 2.105540701 0.144458729 0.000000000

C 0.286303407 -1.368613484 0.000000000

**Complex 6' - MeC(O)OH/MeOCN - HB375_1.010** (0)

C 2.088996400 0.053952526 0.000000000

O 1.727947658 -1.098490085 0.000000000

O 1.229310109 1.087221194 0.000000000

H 0.324001586 0.732778241 0.000000000

C 3.517859214 0.513045574 0.000000000

H 4.179691850 -0.345480947 0.000000000

H 3.705216031 1.131234327 0.876881860

H 3.705216031 1.131234327 -0.876881860

H -1.973579827 -1.966208151 -0.895853265

H -1.973579827 -1.966208151 0.895853265

H -0.416998643 -1.910380642 0.000000000

C -1.462008011 -1.627033881 0.000000000

O -1.441749232 -0.156735294 0.000000000

C -2.591731336 0.420428077 0.000000000

N -3.606203495 0.970139094 0.000000000

**Complex 7' – MeC(O)OH/H_2_O - HB375_1.014** (0)

C -0.681021530 -0.079730445 0.005766635

O -0.074687326 -1.132806626 0.009019329

O -0.080823006 1.111996454 0.012203343

H 0.895181876 0.971974145 0.015914127

C -2.177760208 0.029957279 -0.008482472

H -2.621201172 -0.959051364 0.014511782

H -2.512713221 0.613442580 0.847805271

H -2.495080505 0.560728752 -0.905233617

O 2.420705522 0.032688049 -0.059350450

H 3.029973105 -0.054834180 0.677185916

H 1.813058638 -0.727700486 -0.012605175

**Complex 8' - MeC(O)OH/MeOH - HB375_1.008** (0)

C -1.202626030 -0.080733881 0.001350480

O -0.611284810 -1.138047894 0.102443058

O -0.600049589 1.105867986 0.083328024

H 0.366456693 0.954392657 0.237092681

C -2.682044803 0.036750991 -0.224321922

H -3.120790513 -0.949724025 -0.323201190

H -3.136152092 0.564136745 0.613777977

H -2.873672517 0.626809518 -1.119219604

O 1.846592374 0.056329751 0.496678526

H 1.261812598 -0.716893884 0.421086802

C 2.874600813 -0.021464382 -0.486130393

H 3.460608494 0.891236829 -0.415199693

H 3.530754359 -0.873792439 -0.302082668

H 2.463382464 -0.099629349 -1.495307729

**Complex 9' - MeC(O)OH/Pyridine - HB375_3.011** (0)

C 2.645344034 0.092741541 0.000000000

O 2.164740875 1.205565501 0.000000000

O 1.924083836 -1.025563991 0.000000000

H 0.943439493 -0.802865600 0.000000000

C 4.126364856 -0.182849252 0.000000000

H 4.677116166 0.751395124 0.000000000

H 4.391159424 -0.772422422 -0.876841441

H 4.391159424 -0.772422422 0.876841441

H -3.846646149 -1.674992807 0.000000000

H -1.454803454 -2.351395596 0.000000000

C -3.371016786 0.429145954 0.000000000

C -3.068861934 -0.926050004 0.000000000

C -1.735234863 -1.306055118 0.000000000

N -0.734075053 -0.425148982 0.000000000

C -1.024735679 0.877912953 0.000000000

C -2.331084428 1.347354117 0.000000000

H -0.171166800 1.542776752 0.000000000

H -2.522053727 2.410088189 0.000000000

H -4.399277603 0.762124300 0.000000000

**Complex 10' - MeC(O)OH/MeC(O)NH_2_ - HB375_1.002** (0)

C 1.958822371 0.063448473 0.000000000

O 1.374415430 1.134060225 0.000000000

O 1.357670707 -1.110782181 0.000000000

H 0.356229651 -1.021533495 0.000000000

C 3.458528481 -0.051128874 0.000000000

H 3.907842909 0.935707469 0.000000000

H 3.782529378 -0.610419823 -0.876633742

H 3.782529378 -0.610419823 0.876633742

H -2.066717015 2.014776660 0.000000000

H -0.452642301 1.325455087 0.000000000

H -4.000747045 0.794247112 0.000000000

H -3.780948923 -0.733035461 0.877448542

H -3.780948923 -0.733035461 -0.877448542

O -1.269970702 -1.036578186 0.000000000

C -1.980336270 -0.026663092 0.000000000

N -1.468541231 1.210838117 0.000000000

C -3.483034696 -0.162187009 0.000000000

**Complex 11' - MeC(O)OH/MeC(O)OH - HB375_1.059** (0)

C 1.912545987 -0.057780332 0.000000000

O 1.238974174 -1.078933568 0.000000000

O 1.403277277 1.156430941 0.000000000

H 0.402636075 1.121789389 0.000000000

C 3.412740911 -0.073587659 0.000000000

H 3.775203505 -1.095241731 0.000000000

H 3.782363765 0.456370691 -0.876751219

H 3.782363765 0.456370691 0.876751219

H -0.402636075 -1.121789389 0.000000000

H -3.782363765 -0.456370691 0.876751219

H -3.775203505 1.095241731 0.000000000

H -3.782363765 -0.456370691 -0.876751219

O -1.238974174 1.078933568 0.000000000

C -1.912545987 0.057780332 0.000000000

O -1.403277277 -1.156430941 0.000000000

C -3.412740911 0.073587659 0.000000000

**Complex 1I - NH_3_/CN^-^ - IHB100_10.001** (-1)

N -0.828252957 1.807914375 -1.205489677

H -0.418648109 0.952789009 -0.796174458

H -0.371406840 2.575128170 -0.723670851

H -1.789697494 1.820208123 -0.881549666

C 0.347015050 -0.726530209 0.355711057

N 0.716545948 -1.570224569 1.073493894

**Complex 2I - NH_3_/NC^-^ - IHB100_09.002** (-1)

N -1.142582202 1.718184738 -0.695531665

H -0.587124063 0.870839461 -0.504389511

H -0.642731891 2.471805508 -0.236024105

H -1.998530171 1.599216958 -0.164361672

C 1.053282281 -1.579184385 0.700346807

N 0.472319443 -0.720569467 0.160484396

**Complex 3I - H_2_O/NC^-^ - IHB100_06.003** (-1)

O -0.191375339 -1.365037704 -1.407361054

H -0.103609683 -0.590928264 -0.786452397

H -0.135943946 -2.113683698 -0.810934482

C 0.192839222 1.320457657 1.427471290

N 0.070583765 0.622288025 0.499236875

**Complex 4I - H_2_O/CN^-^ - IHB100_07.002** (-1)

O 0.220311970 -1.715180308 -1.182816955

H 0.127239907 -0.834508093 -0.716063691

H 0.137658852 -2.338231956 -0.458174070

C -0.074650273 0.694596054 0.378790432

N -0.206743139 1.592263011 1.110970151

**Complex 5I – MeOH/CN^-^ - IHB100_07.001** (-1)

O 0.679371003 -1.382309636 0.100133184

H 0.571468069 -0.383993132 0.078073590

C -0.607682953 -1.927592746 0.024192146

H -0.539829132 -2.958594595 -0.338375073

H -1.116979480 -1.951922504 1.000111086

H -1.251750787 -1.366055671 -0.662977985

C 0.117456791 1.393635224 -0.036491418

N -0.187700793 2.515883292 -0.109366222

**Complex 6I - H_3_O^+^/MeOCN - IHB100_01.001** (+1)
O -1.521483378 -1.716488413 0.495043660

H -1.062363600 -2.447333837 0.942523180

H -0.926219885 -0.836209544 0.371590833

H -1.946441714 -2.027010922 -0.322361388

H 0.510012277 1.894428823 1.410365802

H -0.493386401 2.363682951 -0.011997979

H -1.255966283 1.560349659 1.403560466

C -0.359529604 1.665111175 0.805618626

O -0.138893094 0.309285630 0.202853734

C 0.902667974 0.172855910 -0.575150207

N 1.803516474 -0.004241795 -1.267707137

**Complex 7I - NH_4_^+^/MeC(O)H - IHB100_02.001** (+1)

N -0.005156717 0.027401505 2.669644732

H -0.003019297 0.057513514 1.594149372

H 0.822944025 0.490411135 3.040976543

H -0.003677188 -0.946025893 2.972823075

H -0.836842838 0.486776067 3.037470832

H 0.876949172 0.141570135 -2.886403071

H -0.865519769 0.143725264 -2.889663531

H 0.002769056 -1.295930326 -2.270171674

H 0.003886921 1.516355336 -0.963077968

C 0.004233979 -0.212287678 -2.330581632

C 0.002489355 0.415707479 -0.993322912

O -0.000371403 -0.214054866 0.053436712

**Complex 8I – MeC(O)OH/NC^-^ - IHB100_06.001** (-1)

C 1.104710407 -0.400343921 -0.015744828

O 0.698586684 -1.523372021 -0.250774055

O 0.381432798 0.675215139 0.148506994

H -0.686716691 0.563026453 0.069119192

C 2.593893917 -0.105478830 0.121727587

H 3.170886390 -1.014945375 -0.020066651

H 2.891546464 0.641775758 -0.614122034

H 2.797463442 0.313521037 1.107368931

C -3.310000167 0.818801309 -0.025203000

N -2.154898580 0.664363938 0.008559038

**Complex 9I - NH_4_^+^/MeOCN - IHB100_03.002** (+1)

N -0.148694578 -3.409567599 0.000000000

H -0.087922926 -2.332489211 0.000000000

H 0.789060482 -3.807883208 0.000000000

H -0.645553072 -3.729173637 -0.829916654

H -0.645553072 -3.729173637 0.829916654

H -1.155991984 2.496429278 0.900143993

H -0.059062243 3.589107808 0.000000000

H -1.155991984 2.496429278 -0.900143993

C -0.566468541 2.634032503 0.000000000

O 0.532600407 1.639986696 0.000000000

C 0.239468919 0.417855872 0.000000000

N 0.033668568 -0.717961690 0.000000000

**Complex 10I - H_2_O/OH^-^ - IHB100_05.012** (-1)

O -0.078719960 -0.924127744 -0.811003191

H 0.031689558 -0.043993800 -0.081842430

H -0.295773036 -1.654797588 -0.228477884

O 0.139006368 0.942014028 0.786271343

H -0.692705568 1.414923079 0.702832688

**
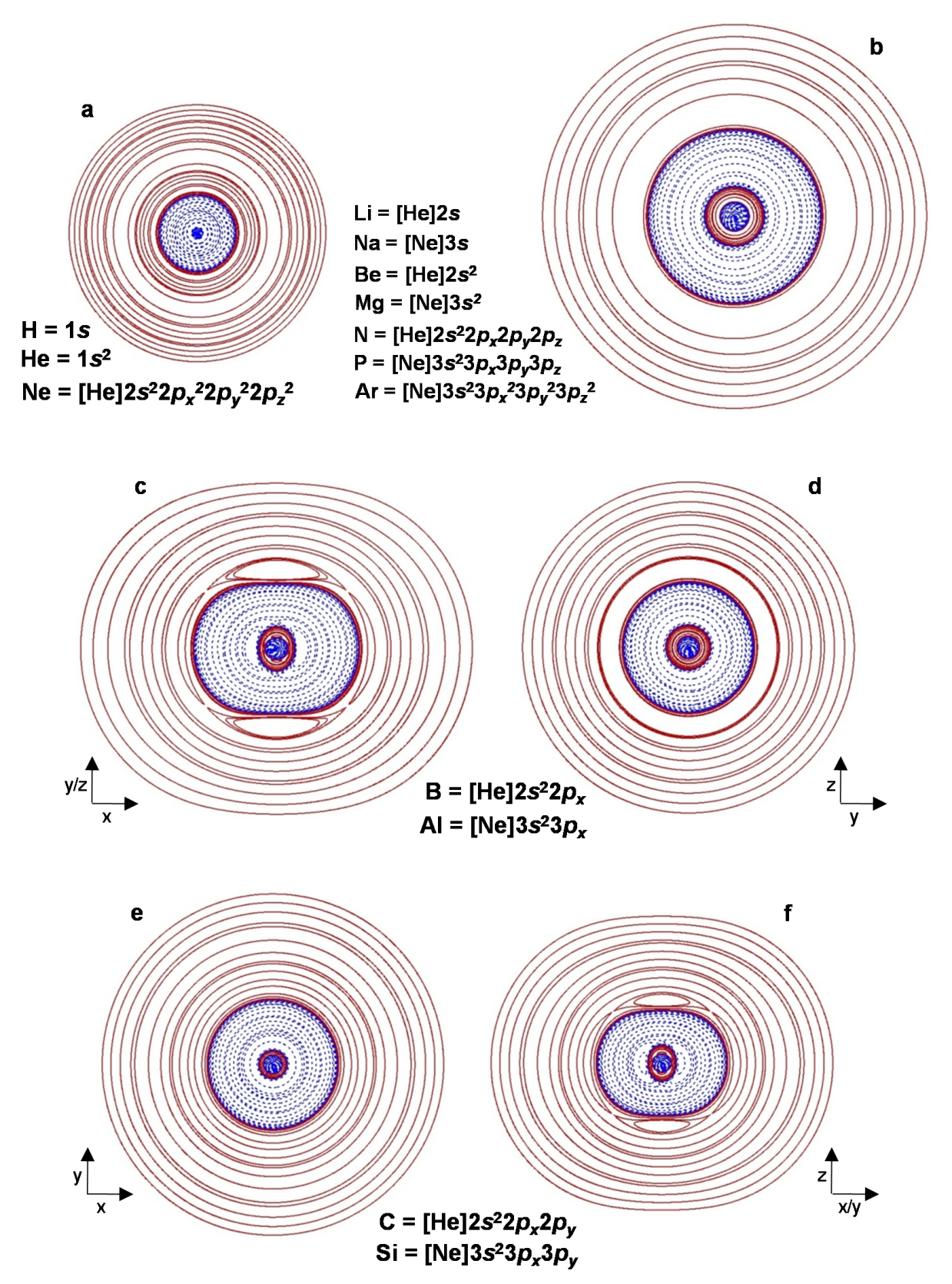
**

**FIGURE S1** 2D-plots of the *H*(***r***) of atomic species showing the partition of the space into *H*^-^(***r***) (dashed/blue lines) and *H*^+^(***r***) regions (solid/brown lines). For atoms featuring four regions in the sequence *H*^-^(***r***)/*H*^+^(***r***)/*H*^-^(***r***)/*H*^+^(***r***), the populations of the inner and outer *H*^-^(***r***)/*H*^+^(***r***) pairs account, respectively, for the core (*ca*. 2 *e* for the first-row and *ca*. 10 *e* for second-row elements, respectively) and the valence electrons.

**
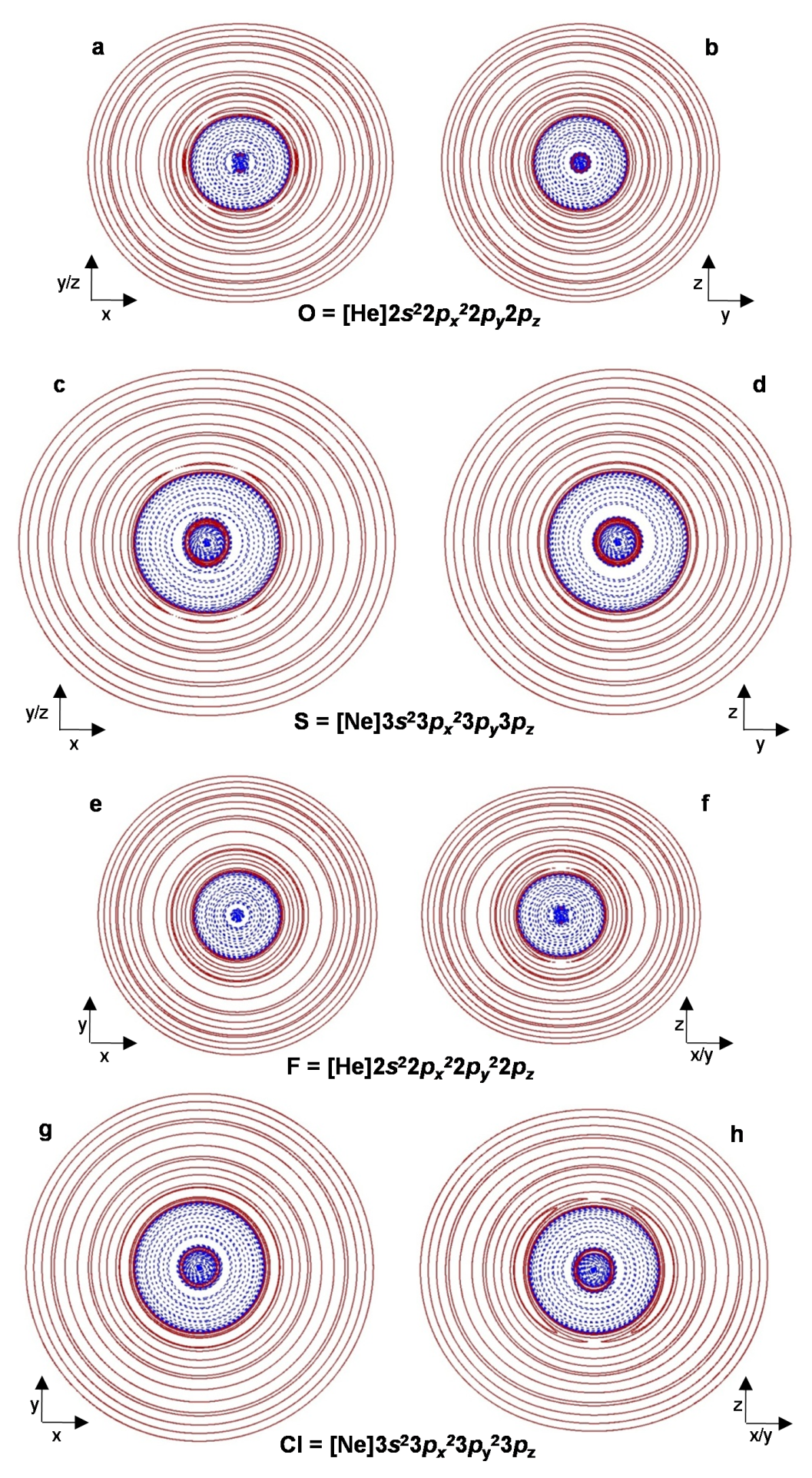
**

**FIGURE S2** 2D-plots of the *H*(***r***) of atomic species showing the partition of the space into *H*^-^(***r***) (dashed/blue lines) and *H*^+^(***r***) regions (solid/brown lines). For atoms featuring four regions in the sequence *H*^-^(***r***)/*H*^+^(***r***)/*H*^-^(***r***)/*H*^+^(***r***), the populations of the inner and outer *H*^-^(***r***)/*H*^+^(***r***) pairs account, respectively, for the core (*ca*. 2 *e* for the first-row and *ca*. 10 *e* for second-row elements, respectively) and the valence electrons.

**
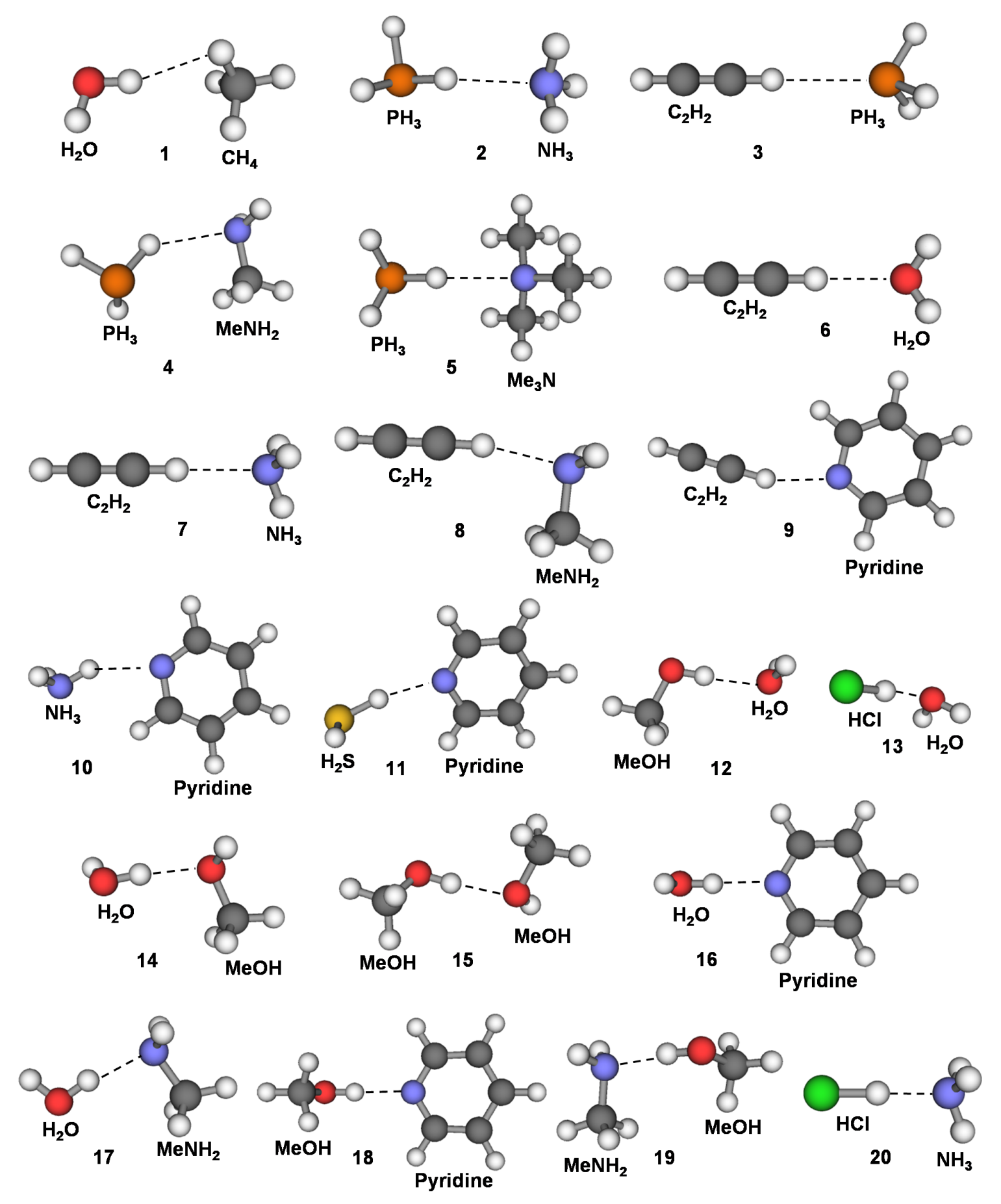
**

**FIGURE S3** Neutral singly-coordinated hydrogen-bonded complexes taken from the NCI Atlas data set [76,77].

**
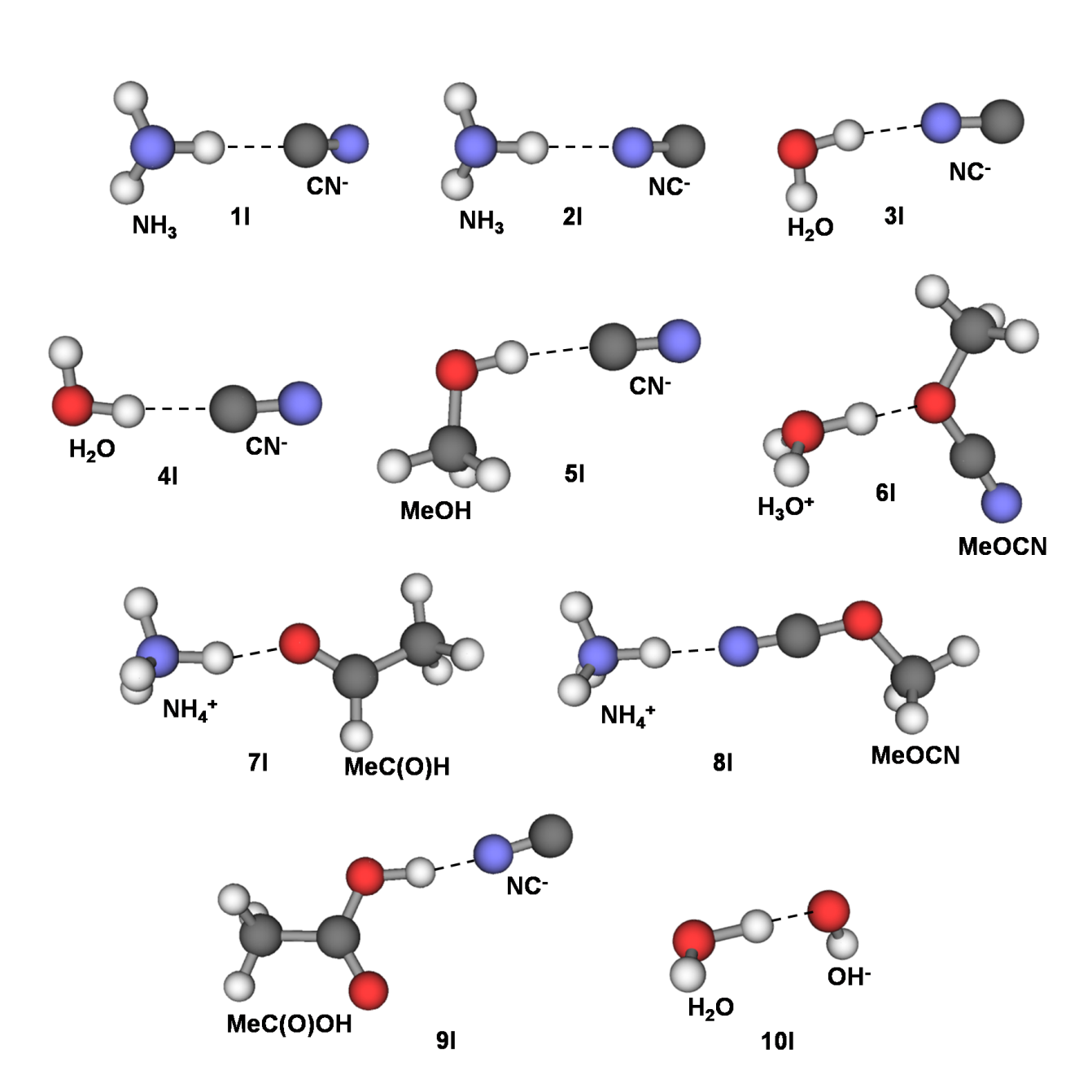
**

**FIGURE S4** Ionic singly-coordinated hydrogen-bonded complexes taken from the NCI Atlas data set [76,77].
